# Social learning under acute stress

**DOI:** 10.1371/journal.pone.0202335

**Published:** 2018-08-22

**Authors:** Lubomír Cingl

**Affiliations:** Department of Institutional, Environmental and Experimental Economics, Faculty of Economics, University of Economics in Prague, Prague, Czech Republic; Universitat Jaume I, SPAIN

## Abstract

Individual decisions are often made simultaneously under social influence and acute stress, yet despite its importance, it has been largely unknown how stress influences the weight which people place on others’ decisions. To answer this I ran a laboratory experiment where 140 subjects were exposed to an acute stressor or a control procedure, immediately before and after which we tested their behavior in a simple Bayesian-updating task. Using three measures (cortisol, heart-rate and mood questionnaire) I show that subjects in the treatment group were under considerable levels of stress. Although stress was expected to increase the weight they put on information coming from the observation of others, I see no effect of stress on subjects’ behavior, either after private or public signals, or on the precision of the updating behavior. This holds across different specifications and after the addition of various personal controls, including the Big-Five personality traits and the psychological measure of conformity.

## 1. Introduction

The effects of social interaction on individual decision making are ubiquitous—be it the "lemming-like" behavior of investors in financial markets, teenagers’ experiments with illicit drugs, conforming to peers at school, or purchase behavior according to fads, fashion and top-10 lists [1–3]. Moreover, choices in social context are often made under acute stress. Traders, lawyers, politicians, and other professionals as well as many ordinary people regularly have to make decisions under severe pressure while being exposed to the social influence of others, for example when deciding in a group. Despite its importance, how stress affects the way people learn from social interactions is still largely unknown. Consider for example a bursting bubble in a financial market, when traders are in a situation where they need to quickly decide about enormous amounts of money on the basis of information from either objective sources like a technical analysis, or from what all the other traders are doing. If they tend to be influenced more by others’ behavior in the times of stress, thus overreacting to the stimuli and deepening the crisis [4], it may bring huge consequences: since their trading behavior influences world market prices, which are crucial for the stability and growth of economies, their overreactions eventually affect the wellbeing of consumers worldwide. Moreover, as an instinctive reaction stress cannot be controlled by human will while it has been shown to seriously affect behavior [5].

This paper is one of the first studies that clearly identify the causal effects of acute stress on individual social learning, although the effects of acute stress on other dimensions of social behavior have already been investigated, including variants of prosocial behavior [6–10], sensitivity to irrelevant social feedback [7], in-group vs. out-group affiliation [8], self-other distinction [11], conformity to higher rank in Navy [12], and the choice of strategy in a duopoly game [13]. Using observational data, the isolation of the causal effect is highly problematic especially in case of studying social learning and conformity as they may be influenced by many confounding factors that humans take into account when deciding whom to learn from, like the role of prestige, kin relationship, and the reputation of success [14,15]. Therefore, I run a laboratory experiment which creates the environment that I have full control over.

The influence of others on one’s decisions resulting in a convergent social behavior has been labeled differently in different disciplines, be it social learning, herding, group-mind, crowd- or mob-behavior, social imitation or mimicry [16]. In economics this phenomenon has mostly been studied in the context of herding in financial markets [17–22], online-product choice [23,24], peer effects [25] and social learning [26,27]. The peers’ influence on decision-maker can generally be divided into two channels [25,28]: (i) direct learning from observing the peers’ choice (*social learning*) and (ii) a direct utility gain from the same (or similar) behavior (*social utility*). The *social learning* channel consists of just a bounded rational use of information a decision-maker obtains from the observation of the decisions of others and all behavioral biases are neglected. The *social utility* channel can be extended to comprise not only the natural preference for conformity [29,30], but also various social and psychological factors such as personality type [31,32] while it excludes any learning factors. As an example of what personal attributes may particularly matter, Baddeley et al. [33] identified that a younger female with high scores in the personality traits venturesomeness and impulsivity has a higher probability of following others in her decisions. In this paper I focus solely on the *social learning* channel while controlling for the role of selected personal attributes.

Paradigms used in the experimental literature for the measurement of social learning range from a simple prediction of judgment of others [34], a multiple-cue probability learning paradigm [35], to settings testing different learning strategies in Bayesian-updating processes [36]. In economics, information cascades hold a prominent place due to having a solid theoretical background [2,37–41] and having been extensively experimentally [42–44] and empirically [20,45] tested. In the information cascades setting, a series of individuals make a binary decisions over an unobserved state of the world based on imperfect information private to them, and on the observation of decisions made by others earlier in the sequence.

To test the representativeness heuristics [46] against the predictions of Bayes’ rule, Grether [47,48] designed an even simpler probabilistic task that unlike the information cascade setting does not contain the element of making decisions in a sequence. It is useful as it allows for a precise calculation of the optimal reaction to a new signal (information updating) according to the Bayes formula, but does not contain any other features that would potentially complicate the decision making procedure of subjects. It has been frequently employed in the literature to test deviations of human updating behavior due to various psychological biases, like apart from the above mentioned representativeness heuristics, the reinforcement heuristics [49], confirmation bias [50], general behavioral strategies [51], the role of reaction time [52], and has been even used as a suggested task for teaching the Bayes’ rule in the classroom [53]. Therefore I considered this task to be optimal for the purpose of my study.

In my operationalization of Grether’s task, subjects state subjective probability of which of the two unobserved states of the world is more likely to have occurred, and repeatedly restate it based on a series of first private and then public signals. They are incentivized in each decision to make a correct prediction. The structure and intensity of the signals differ across rounds but are the same for the treatment and control subjects in a given round. As has been shown that learning under stress may be impaired [54,55], I implemented a series of rounds before and after the treatment manipulation, which leads to a difference-in-differences econometrical approach.

The task setting allows for the examination of the difference between the treatment and the control group along several dimensions. The main aim of the study is to estimate the stress-induced difference in social learning that is captured by the updating behavior after receiving a public signal. Apart from that, I also examine the stress effect on general updating behavior that is revealed by the behavior after the private signals, and the general precision of subjectively stated probabilities compared to the predictions calculated using Bayes’ rule. As the control variables I employ the Bayes prediction that allows me to compare the reactions to the same strength of signal, personality profile as measured along the Big-5 dimensions [56,57], conformity [58], gender and age.

I work with the definition of acute stress as the physiological, psychological and behavioral reaction arising from perceived environmental demands threatening an important goal of an organism [59,60]. Generally speaking, an individual’s reaction to stress seems to be highly complex and to differ with respect to the type and duration of stressor [61] as well as with respect to various individual characteristics [62,63] and has been subjected to a number of models [60,64,65]. To induce acute stress I employ the Trier Social Stress Test for Groups [66,67] which consists of a stress-inducing or a control procedure that differ only in the stressful aspects, while cognitively are almost identical. The type of stress it induces in subjects, the acute psycho-social stress, seems to be the most common type of stress experienced by the general public in the workplace which enhances the external validity of the results in contrast to manipulations inducing a physical or pure psychological stress [65,68]. The peak of the cortisol response occurs 20 to 40 minutes after the start of the stressor and the whole cortisol response lasts 40 to 60 minutes after the stressor ceases [69,70]. I use this fact in the experimental design when the behavioral task is administered right after the end of the stress procedure when the stress response should be the highest and should last over the whole decision-making procedure.

As to studies focusing on social learning under stress, conformity under stress has already been studied but stress was induced rather indirectly: the authors experimentally tested conformity within the ranks of male U.S. Navy students under the threat of tear-gas infusion into the room [12]. Both the high and low status subjects behaved in a similar manner and became more willing to accept the opinion of their partner. Unfortunately, the authors do not provide any evidence that the subjects were under stress and their measure of conformity is rather crude. In another study subjects were exposed to time pressure while their cortisol and heart rate were measured [13]. Such manipulation is normally used in order to produce intuitive responses rather than stress. The authors argue that because the cortisol levels slightly increased, their manipulation was successful and they generally find more imitation relative to control. Lastly they show that subjects with higher physiological responses are more likely to imitate the choices of others, though with no difference across the treatments.

I hypothesized that stress would affect the social learning channel and people would become more receptive to the social information. Evolutionarily, stress seems to have developed to enhance the chance of survival that in the case of a collective species is closely connected with the survival of others. Coordinated actions among the group, like in case of fight-or-flight reaction [71] escaping a predator or fighting it collectively should increase the chance of survival of all members of the group [72,73]. I study if this instinct spills over to the decision making in a rather abstract context, which could however be then well generalized to other areas of decision making. One of the physiological effects of acute stress is the down-regulation of prefrontal cortex activity [63,74–76]. Prefrontal cortex is generally known to be the centre of executive and cognitive control and willpower [77,78], particularly the medial part of prefrontal cortex is associated with social behavior as has been shown in a number of autism studies [79] and studies using fMRI [80–82]. With the expected deterioration of higher cognitive abilities I hypothesized that the precision of the reaction to a new signal would decline under stress. Apart from the precision of estimates, the experimental design also allows for investigation of general updating behavior from objective sources that may also be affected due to the deterioration of cognitive abilities. Stress may further cause a shift in risk-preferences [74,83–85] which may demonstrate in generally more conservative strategies, although the literature is far from any consensus on the effects of acute stress on risk-preferences. Moreover, other behavioral and psychological traits important to herding behavior may be affected by stress as well: even though personality traits are considered stable under stress [67], their interaction with stress may produce a situation when the resulting behavior is different for individuals high or low in the particular trait, such as in case of trait anxiety and competitive confidence [86]. Here I measure the Big-Five personality traits [87] and a conformity profile to capture such possible effects. Last, at least two related studies examined the rate of imitation under high levels of time pressure to find signs of more imitation [13,88]. Because time pressure may induce stress and its effect is then combined with lack of time making subjects decide intuitively, this evidence point in the direction of finding more social influence under stress.

## 2. Materials and methods

### 2.1. Task: Bayesian updating

In this section I describe in detail the task I use for the measurement of Bayesian updating that is based on Anderson and Holt [42] and Grether [47,48]. Since the task is quite complex, I first describe the general procedure in a given round, next I introduce the payment procedure as well as its pros and cons, and lastly I explain the structure of public signals. For the precise instructions given to the participants please refer to the Supplementary Online Material (SOM) S3 File.

**Fig 1:**
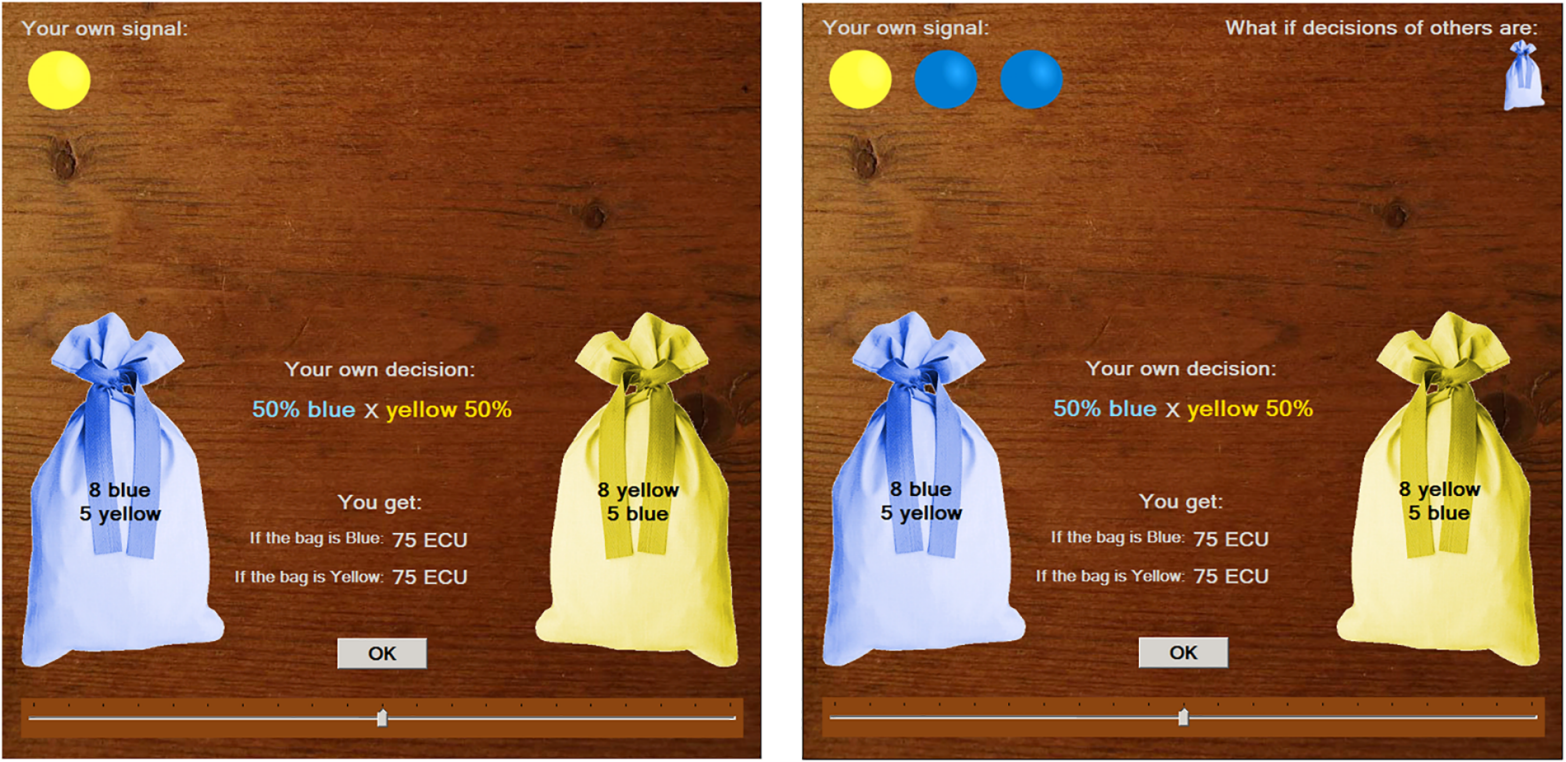
The layout of the decision-making environment. *Note*: The representation of the decision-making environment of the subjects. The left part of the Fig shows the decision after the arrival of the first private signal (top-left corner). The right part of the Fig shows the representation of public information in the upper right corner; particularly in the strategic form ("What if" scenario).

In the task, subjects state their subjective beliefs about which of the two possible states of the world occurred based on signals they receive, and are monetarily rewarded for a correct prediction. The states of the world are framed as two possible unmarked bags containing marbles of two colors, blue and yellow. The “Yellow” bag contains more yellow marbles than blue marbles whereas in the “Blue” bag the ratio of the colors is symmetrically reversed. At the beginning of each round, one of the bags is randomly chosen by the computer with a 50% chance; the same bag for all participants in a given round. First of all, subjects are informed about the composition of the colors in both bags. With each new signal, subjects are asked to state or revise their beliefs using a slider that indicates a percentage probability of both outcomes (visualized in Fig 1). A *private signal* is the information about the color of a ball drawn with replacement from the chosen bag. After receiving several private signals and restating the subjective probability, always revealing color of just one ball at a time, participants are presented with the *public signal* in one of the forms described further. Public signal is essentially information about the decisions of some other randomly chosen players. The parameters (the ratio of the colors in bags as well as the number of private and public signals) varied across the rounds but stayed the same for all subjects in a given round, so that the situations where the public signal could overturn the content of the private signals (Table 1) would be maximized and also not a simple heuristics for the decision making could be developed.

**Table 1:** Round structure: Number of balls in the "Blue" bag.

Round number	Number of blue balls	Number of yellow balls	Number of private signals	Players per group	Type of public signal	Strategy method: Order of public signals
trial 1	10	3	2	4	Direct response	
trial 2	8	5	3	3	Strategy method	Real
round 1	7	2	2	4	Strategy method	Opposite signal first
round 2	10	7	2	5	Direct response	
round 3	5	4	2	3	Strategy method	Real
round 4	10	5	4	4	Direct response	
round 5	13	4	3	3	Strategy method	Opposite signal first
round 6	7	2	3	3	Strategy method	Real
round 7	13	4	2	4	Direct response	
round 8	5	4	3	2	Strategy method	Opposite signal first
round 9	7	2	4	2	Direct response	
round 10	10	7	3	3	Strategy method	Real
round 11	5	4	3	3	Direct response	
round 12	13	4	4	2	Strategy method	Opposite signal first
round 13	10	5	3	3	Strategy method	Opposite signal first

*Notes*: The trial rounds were not incentivized. The treatment intervention was implemented between the round 5 and 6.

At the end of each round, subjects receive feedback on which bag was really chosen by the computer and how much ECU this round would pay if selected for payoff. Subjects were paid for each decision according to the quadratic scoring rule (QSR) for stating their beliefs in a given round, and all decisions in a given round added up to a total amount of ECU which could be earned from this round. In order to ease the cognitive demand required by understanding the rule [89], the QSR was explained and demonstrated by a table with selected probabilities and respective payments in the instructions. Moreover, as they moved the slider, the respective amount paid in case one of the bags was chosen changed in real time [90]. Prior to starting the task, subjects were informed that out of the 13 rounds, three would be randomly chosen for payoff at the end of the experiment.

QSR is one of very frequently applied methods for the incentivized elicitation of subjective probabilities [91]. The main concern is that it is incentive compatible only if the subjects are risk-neutral, which is commonly not the case as they are mostly risk-averse [92]. A risk-averse agent may want to hedge away from the extreme outcomes in order to minimize losses [93]. There are several methods that would allow the QSR under non-risk-neutrality to be truth-telling, such as paying small stakes, randomize payments or estimate the deviations from risk-neutrality (see e.g. review in [91]). However, for the purpose of a comparison of treatment intervention on two groups randomly created from the same population, we can reasonably assume that the distribution of risk-preferences and consequently the bias introduced by using QSR is on average the same in both of them. A potential confounding factor may arise if the treatment intervention changes also risk-preferences. For this purpose, an independently incentivized risk-elicitation task was implemented after the end of the Bayesian updating task (see the next section) and the potentially created confound is addressed in the Results section and in a detailed discussion in Section B in S1 File in SOM.

In each round after all private signals had been revealed, a public signal was presented. The public signal was conveyed by showing on-screen a small bag in the upper right corner of a blue or yellow color (see right part of Fig 1). The color of the small bag represented the color of the bag the other subject indicated to be more probable, rather than their point estimate. If the odds were 50/50, the color of the small bag was chosen randomly. One small bag indicated decision of one randomly chosen other participant. Participants either saw the color of the bag(s) and made a direct response, which was framed as "Scenario: Reality", or were presented with all possible combinations of the actions of others and were asked to state the probability conditionally for each situation. This strategy method was framed as the "Scenario: What if" and participants were paid only according to the situation that really occurred. Here also the order of the public signal was manipulated so that in five rounds the opposite signal to the current beliefs of participant was presented first, whereas a random order of the signals was implemented in another three rounds (Table 1). The strategy method also allowed for a richer set of observations with a given set of parameters than would be possible with the direct method only.

### 2.2. Task: Risk-preferences

To measure risk-preferences I used a standard multiple-price list task after Dohmen et al. [94] which was computerized using Z-Tree [95]. Subjects choose in ten situation either a safe payment or a lottery with equal probabilities of winning and not winning a fixed amount of 4000 ECU, and the safe payment starts at 0 ECU and gradually increases between the situations in steps of 300 ECU up to 2700 ECU. Subjects were informed that one situation would be selected at random and would be paid according to their decision in the selected round. A subject with consistent risk-preference profile should start with choosing lottery if the safe payment is 0 and at some point switch to the preference of the safe payment. This switching point is directly informative of the individual certainty equivalent and can be used as a proxy for the risk-profile of a participant. I allowed for an inconsistent behavior by allowing subjects to choose freely in any pair of actions. The effect of treatment manipulation on risk-preferences is discussed in a different article [96] where also the detailed instructions and the screenshot of the task can be found, while here the task serves only for the purpose of a robustness check.

### 2.3. Treatment manipulation: Stress-inducing procedure

Subjects were exposed to a slightly modified Trier Social Stress Test for Groups (TSST-G, [66]), which is a standardized psychological protocol for inducing acute psychosocial stress. The changes to the original protocol concerned mainly the information given to participants regarding the behavioral training of the panel members and regarding the fact that the video recordings would later be analyzed, so that no deception was present. The approval of the Institutional Review Board of the Laboratory of Experimental Economics, Faculty of Economics, University of Economics, Prague (IRB LEE 001–2012) was obtained prior to the experiment. The subjects were randomly divided into two groups of seven and after reading the instructions silently for 3 minutes, they went into two separate rooms adjacent to the lab that were set-up for the procedures. The TSST-G protocol consists of two parts: a public speaking and a mental arithmetic part. The stress condition frames these two parts as a mock job-interview and a serial subtraction of 17 from 4578, respectively, while in the control group subjects jointly read a scientific text aloud in a low voice, and said out loud numbers in steps of a certain magnitude, e.g. 5, 10, 15, 20 etc. After entering the room, the participants stood in places marked by their participant numbers, and had headphones with ambient noise on so that they would not hear each other. In the stress condition, two additional experimenters, who were referred to as a "committee" during the procedure, sat at a desk in front of the participants, wore white laboratory coats and had a video camera by their side. The committee had been trained not to give any feedback on the subjects’ performance either verbally or physically. With a neutral expression on their faces, they called subjects in a random order, who then had two minutes to present their job-interview. When all subjects had finished their job-interviews, the committee asked the subjects again one-by-one to complete the arithmetic task for one minute. The committee made notes during the whole procedure. A careful debriefing was carried out with the stress group before they received the payments at the end of the experiment. In the control condition, the two panel members were referred to as "assistants" and subjects were informed that their role was only to measure time and provide instructions for the next part of the procedure.

### 2.4. Sample & procedures

By using ORSEE [97] 140 healthy subjects were recruited: 67 females (mean age 22.1, SD = 2 years) and 73 males (mean age 22.6, SD = 2.7 years). The sessions were run in two batches with identical procedures, experimenters and committee members, therefore I pool the results. The first five sessions were run in April 2012 and the next five sessions in November 2014. Subjects were mostly students of economics, management or related disciplines (72%). I followed best practices in order to avoid any factors confounding cortisol measurement [98]: Subjects received an invitation email already with instructions to abstain from fatty food, nicotine and heavy exercise at least 2 hours prior to the experiment. Immediately before the experiment subjects were screened for any circumstances that could potentially disrupt the cortisol response: health status, drug intake, caffeine, heavy meals, and contraceptive intake. I then performed robustness checks and none of the problematic factors mattered for the main results (available upon request). With one exception the participants were all normal body-weight and twelve women indicated taking oral contraceptives. Out of these twelve women, five were assigned to the treatment group; two of these did not show the expected cortisol increase.

Subjects were not notified about the purpose of the experiment beforehand in order to minimize the selection bias. They signed an informed consent form which referred to the treatment procedure as a "challenge" task and were specifically and repeatedly informed about the chance to leave the experiment at any point in time. None decided to leave before the regular end of the experiment.

All experimental payoffs were denominated in experimental currency units (ECUs) and were converted to Czech crowns at the end of the experiment at a conversion rate 32 ECU = 1 CZK. The experiment lasted on average a little less than 2.5 hours and was conducted in English. All subjects were listed in the database for experiments conducted in English and indicated no difficulties understanding the instructions or speaking. One experimental session included 14 subjects (seven in the treatment and seven in the control group), two experimenters and four members of the committee for the TSST-G procedure. All sessions started at 16:30 in order to make the cortisol measurement comparable across sessions due to the natural diurnal cortisol cycle. The average payoff was 490 CZK, i.e. 19.6 EUR.

**Fig 2:**
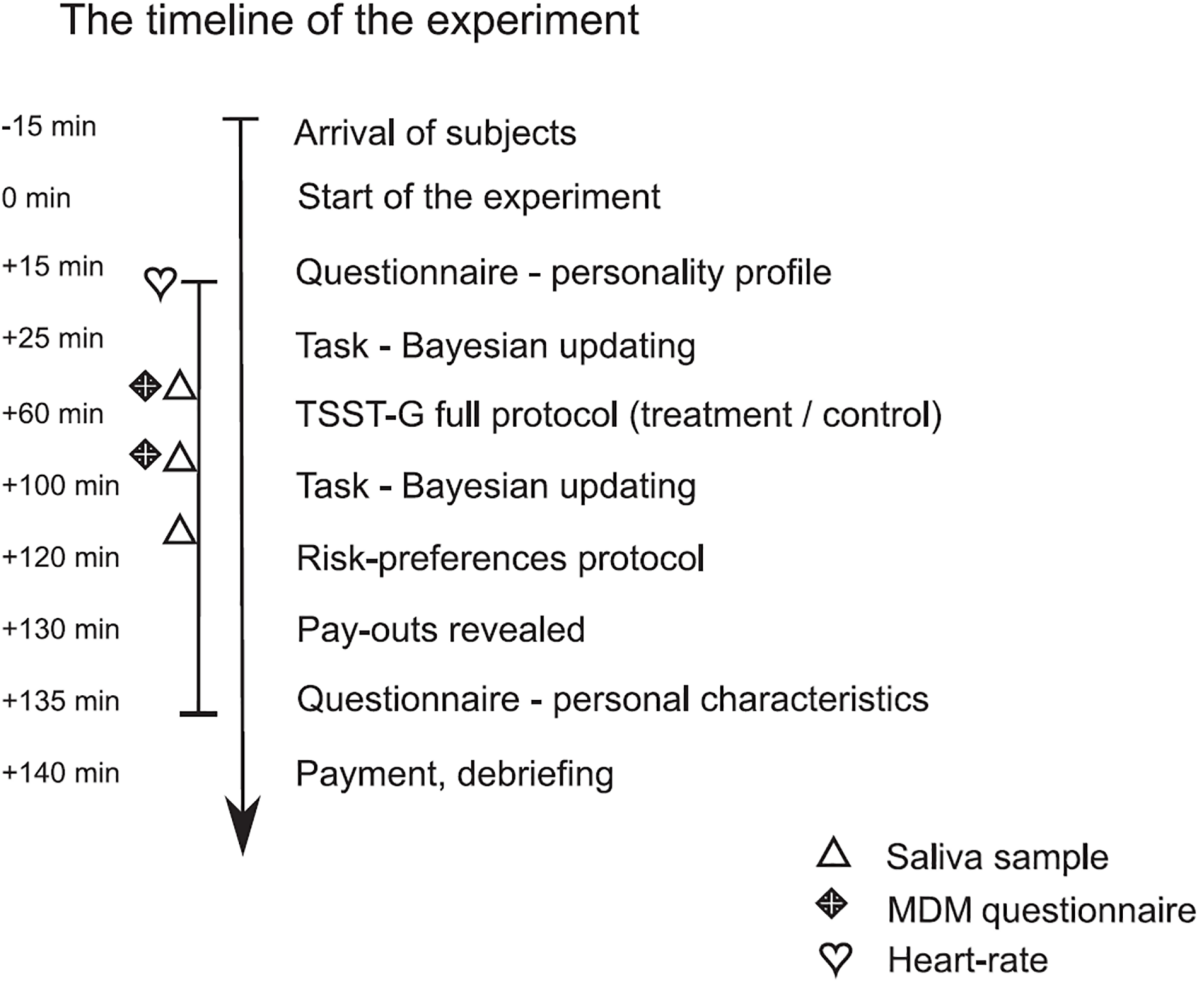
The timeline of the experiment.

The timeline of the experiment is summarized in Fig 2. After arriving at the laboratory subjects were randomly assigned to computers, signed the consent form and the general instructions were read aloud by the experimenter. Participants got the heart-rate monitors attached, and were asked to fill-in an on-screen questionnaire assessing their personality profile. The instructions for the probabilistic task were then read aloud and subjects had to answer three questions confirming their understanding. Then they had two trial and five real rounds of the task. At this point they were asked to give the first sample of saliva and fill in the first part of the MDM questionnaire. Instructions to the TSST-G treatment and control procedure were distributed next. Participants read them silently and had few minutes to either prepare for the job interview or for reading a scientific text which was subsequently performed in adjacent separate rooms, i.e. the full TSST-G treatment and control procedures were carried out. After this, the participants returned to the lab, were seated back at computers, the second sample of saliva was collected and they filled in the second part of the MDM questionnaire. Then the participants were to solve eight more rounds of the previous task. When finished, the participants gave their third saliva sample and completed a separately paid-for task aimed at measuring their risk-preferences. At the end of the experiment, three rounds of the task were randomly drawn for payment, subjects completed a short questionnaire on their personal characteristics and proceeded to payment. All subjects were paid in private, and when the control group had left the laboratory, a thorough debriefing of the TSST-G procedure was carried out with the treatment group. Participants were asked to sign a statement of confidentiality with respect to the experimental procedure.

### 2.5. Questionnaires—Personality measurement

Participants filled in the 50-item set of IPIP Big-Five Factor Markers [57] which is a measure of the five major factors of personality, openness to experience, conscientiousness, extraversion, agreeableness and neuroticism. In addition to that, I included a conformity inventory IPIP measuring construct similar to the revised Jackson Personality Inventory [58] to control for the natural behavioral propensity to conform to the opinion of others.

## 3. Results

### 3.1. Randomization check

I perform a randomization check of observable characteristics that may influence the stated probability after public signals. Table A1 in the S1 File in SOM shows that the treatment and control groups were balanced with respect to gender, age and education. Further factors that may influence the results are the "Big-5" personality traits and conformity. The treatment group was higher in extraversion and neuroticism, while not statistically different in the other dimensions, which is another reason to add these controls to the regression analysis, apart from controlling for the behavioral explanation of the social influence.

### 3.2. Manipulation check

**Fig 3:**
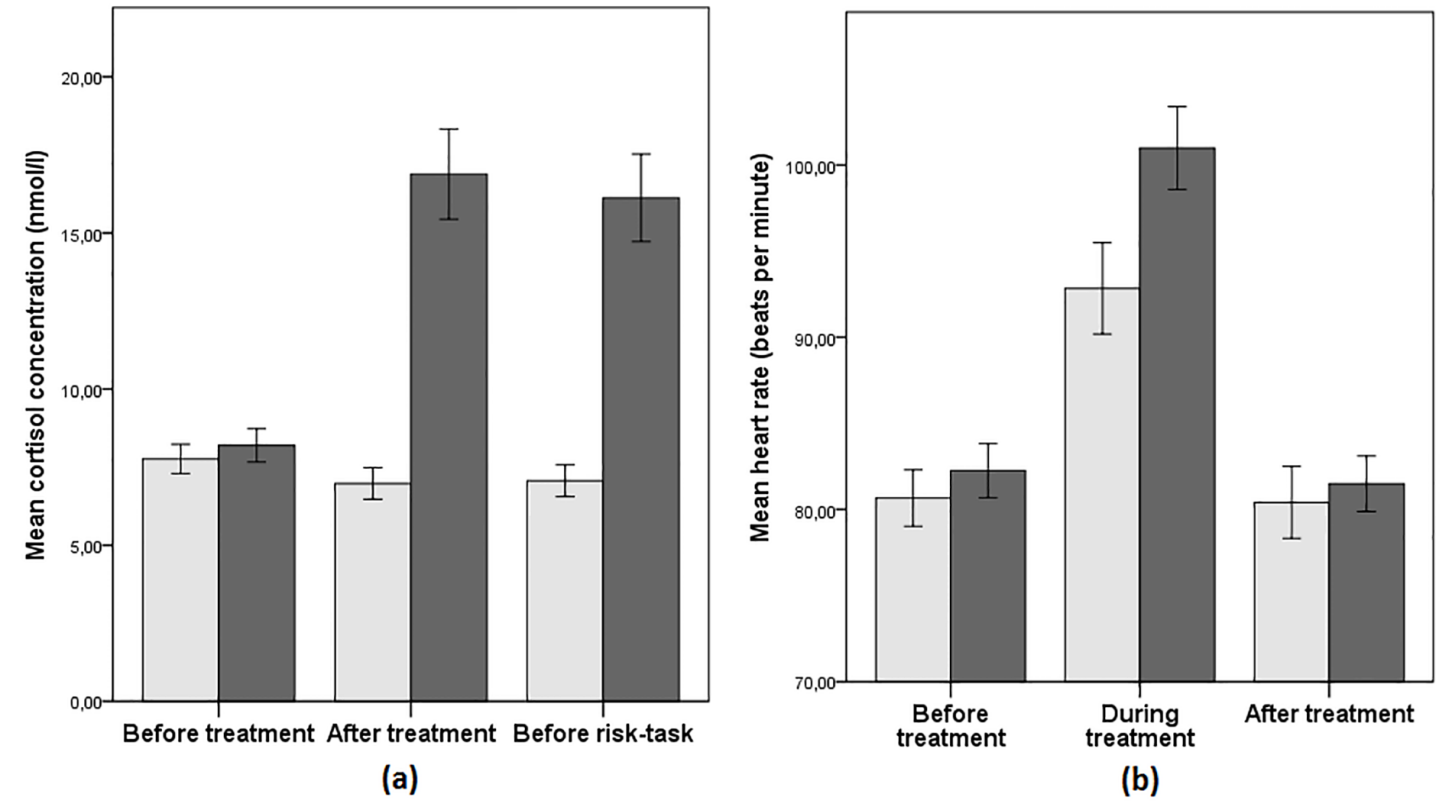
Induced stress reaction: Cortisol and heart-rate. *Notes*: The darker color indicates the treatment group. Panel (a): Mean levels of free salivary cortisol; Sample 1 was collected before the treatment or control TSST-G procedure, sample 2 after the TSST-G procedure and sample 3 before the risk task. Panel (b): Mean levels of heart-rate calculated from individual averages over the time periods before, during and after the TSST-G procedure. Error bars indicate standard errors of the mean.

Using three measures I show that stress induction was successful. First I present in panel (a) of Fig 3 the cortisol reaction that I consider to be the most reliable indicator of induced stress. The level of concentration of salivary cortisol is not different for the treatment and control groups before the TSST-G procedure (two-sample Wilcoxon ranksum test: z = -0.21, p = 0.83, d = -0.1; reported effect sizes are Cohen’s *d*, corrected for uneven groups.), but the sample taken after the procedure as well as the sample taken after the end of the task show a significant increase for the treatment group (z = -6.22, p < 0.001, d = -1.1 and z = -6.05, p < 0.001, d = -1.04, respectively).

Second, panel (b) of Fig 3 summarizes the heart-rate before, during and after the stress procedure. I note that there were technical problems with measurement in several subjects. With some we were completely unable to find the signal while with others the signal kept turning on and off during the procedure; therefore I do not have a full number of observations for this measure. Heart-rate does not differ between the treatment and control groups before (z = -0.77, p = 0.44, d = -0.14) and after (z = -0.99, p = 0.32, d = -0.12) the TSST-G procedure, however, there is a significant difference during the procedure (z = -1.84, p = 0.066, d = -0.41) which supports the claim that subjects in the treatment group were physiologically under stress.

**Fig 4:**
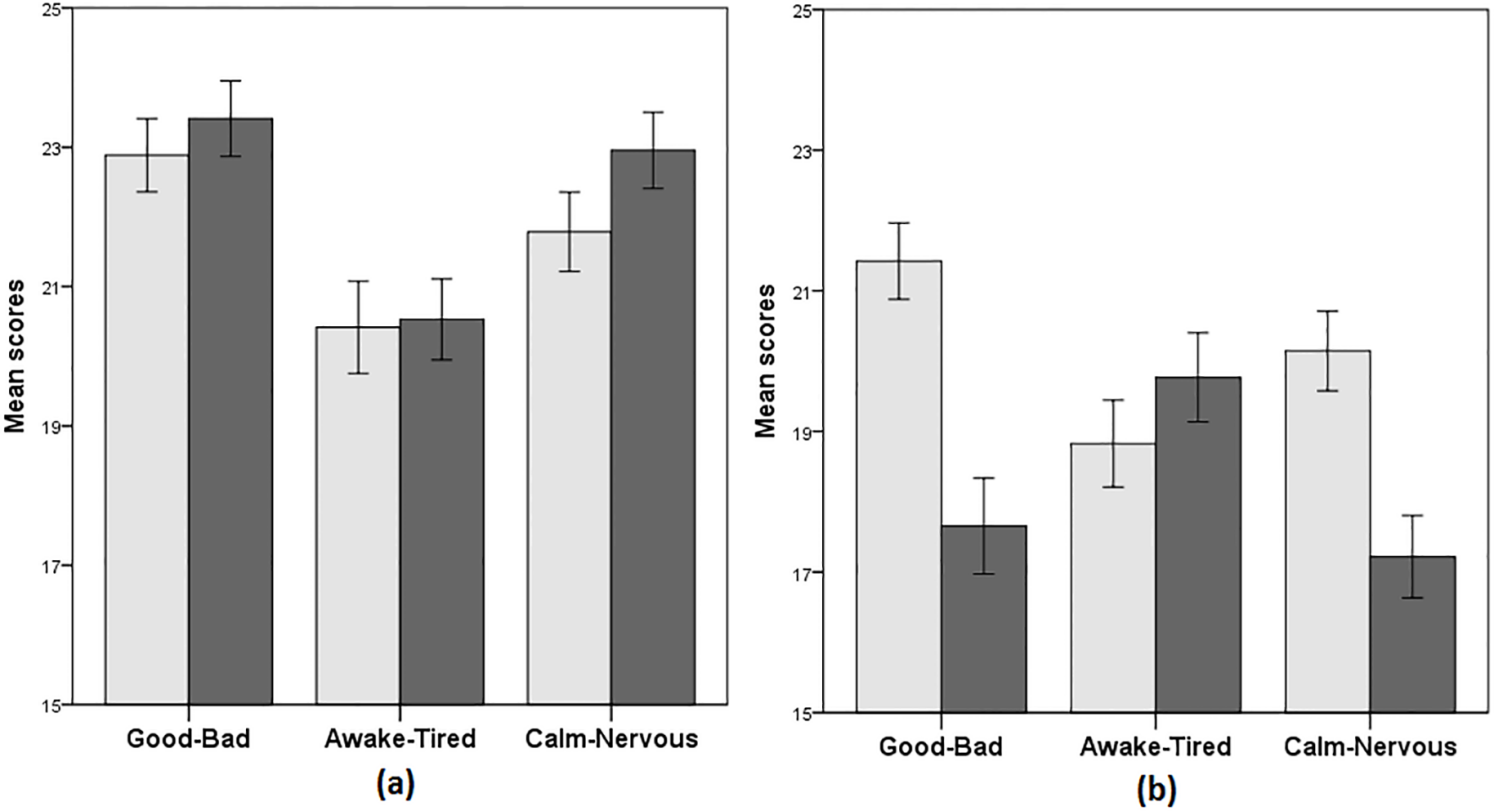
Induced stress reaction: Mood. *Notes*: Mood before (panel a) and after (panel b) the TSST-G procedure—scores from the Multidimensional Mood State Questionnaire. The darker color indicates the treatment group. Error bars indicate standard errors of the mean.

The subjective effect of stress was captured by the change in the subjects’ mood reported in the MDM questionnaire. Panel (a) of Fig 4 shows that before the treatment procedure, the treatment and control groups’ scores were not different in any of the three dimensions ("good-bad": z = -1.05; p = 0.29; d = -0.11; "awake-tired": z = -0.83; p = 0.83; d = -0.00; "calm-nervous": z = -1.2; p = 0.23; d = -1.12). Panel (b) of Fig 4 reveals that after the TSST-G procedure, the treatment group reported scores significantly different than the control group: the treatment group scored closer to the "bad" (z = 3.60; p < 0.001; d = 0.68) and the "nervous" (z = 3.44; p < 0.001; d = 0.61) dimensions. The last "awake-tired" dimension was not different across the two groups (z = -1.59; p = 0.11; d = -0.1). This finding shows that subjects in the treatment group were also under psychological stress. For further details see Table A2 in S1 File with the descriptive statistics of all three variables further divided by gender.

The changes of the two physiological measures (cortisol and heart-rate) are significantly correlated, (ρ = 0.385; p < 0.001). The cortisol response is further correlated with the MDM score in the good-bad dimension (ρ = -0.259; p < 0.01) and in the calm-nervous dimension (ρ = -0.23; p < 0.01). The association of heart-rate and psychological measures is significant in the calm-nervous dimension (ρ = -0.21; p = 0.02) and the awake-tired dimension (ρ = 0.15; p = 0.09). The three mood dimensions are further highly correlated, as could be expected.

### 3.3. Social learning

The variable of interest in the analysis, *Decision*, is the stated probability entered by subjects after each new signal using a slider that they moved from the initial point 50/50. I take the value of the distance from the 50/50 point to the new position of the slider in the direction of the new signal. Apart from the actual decision of the subjects I also calculate the optimal decision (variable “*True*”), given all information a subject had received prior to the current decision in a given round, i.e. the number, direction and strength of all preceding signals. While calculating *True* I assume that a decision maker is rational in that she uses the Bayes formula for updating her priors and disregards any irrelevant information in the sense that there is no interdependence between the answers in the "What-if" scenario and between the rounds. I am aware of the on-going debate as to whether people really use the Bayes formula in their decision making and thus I note that it is a rather simplified assumption made for the sake of convenience. The calculation of *True* was done in the following manner: let’s define *b* the number of blue balls in the Blue bag, y the number of yellow balls in the Yellow bag, and *n* the total number of the balls in the bag. The optimal move after the first signal, which was always private, was calculated by first finding the probability of drawing a blue ball given the composition: Pr(*b*|*B*) = *b*/*n*. The probability of drawing the yellow ball is complementary, i.e. Pr(*y*|*B*) = 1 − *b*/*n*. The value of *True* is the probability of the bag being Blue, given this signal, which is $\text{Pr}\left(B|b\right)=\frac{\text{Pr}\left(b|B\right)}{\text{Pr}\left(b|B\right)+\text{Pr}\left(y|B\right)}$. See Section D in S1 File in SOM for further details about the calculation of *True* for more complicated private and public signals. For the calculation of the public signals I assume that subjects also took into account the possibility that when the other subjects stated exactly the same probabilities of both bags having been selected, the resulting signal was chosen at random.

Next I define a new variable *Difference* as the difference between *Decision* and *True*, which then shows whether subjects over- or under-valued the signal compared to the *True* value, if the *Decision* was higher or lower than the *True*, respectively. Recall that *True* captures the informational content of the signals, therefore also the composition of the bag, number of signals received and the share of signals for the chosen color. Table A3 in S1 File provides the descriptive statistics of the main independent variables used in the analysis.

**Fig 5:**
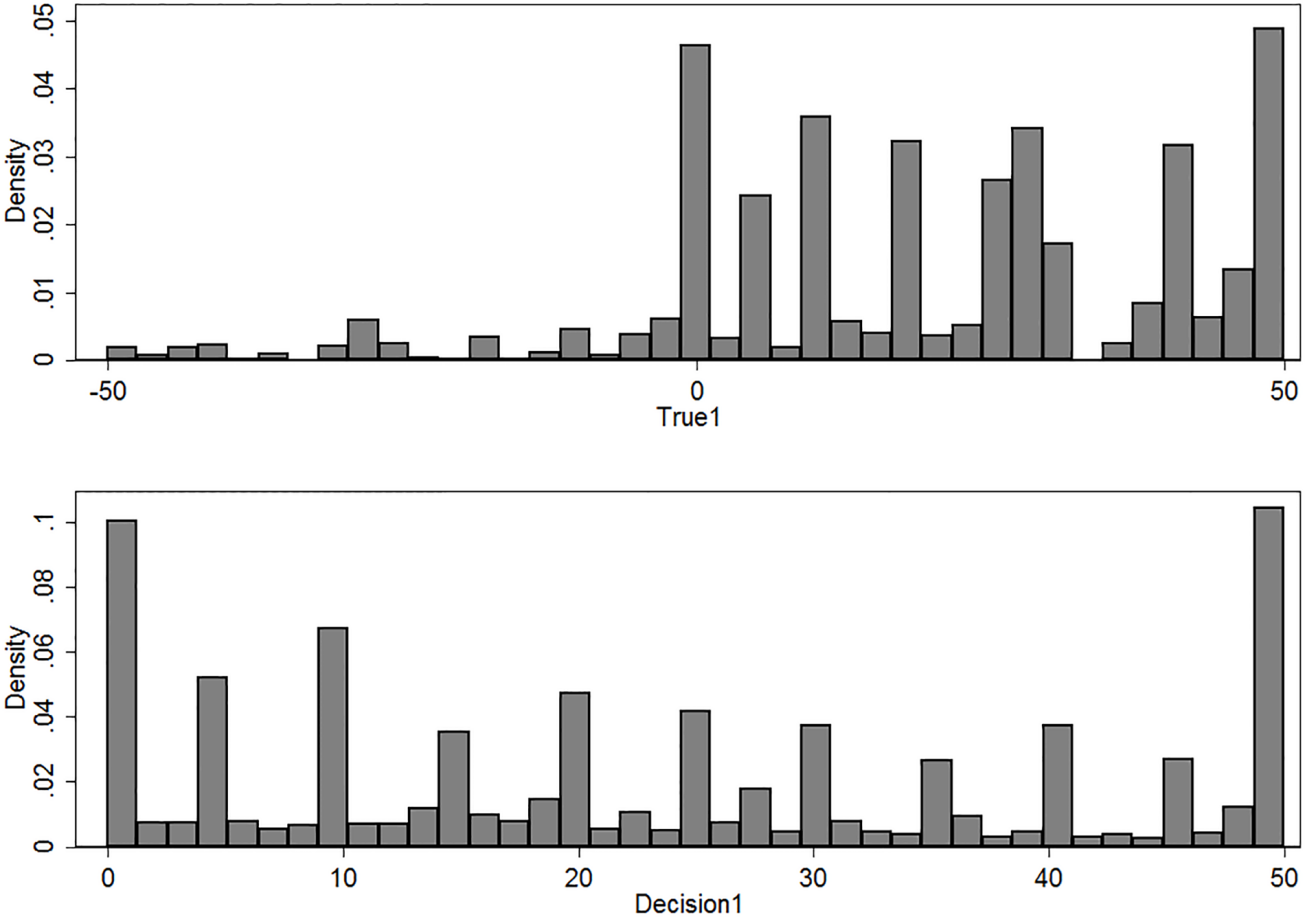
Histograms of variables *True* and *Decision*.

Fig 5 illustrates that subjects suffered from typical behavioral biases identified in the literature, such as stating more likely probabilities rounded to 5 or 10 and being reluctant to state the probability closer to the extreme, when contrasted with the *True* probability.

**Fig 6:**
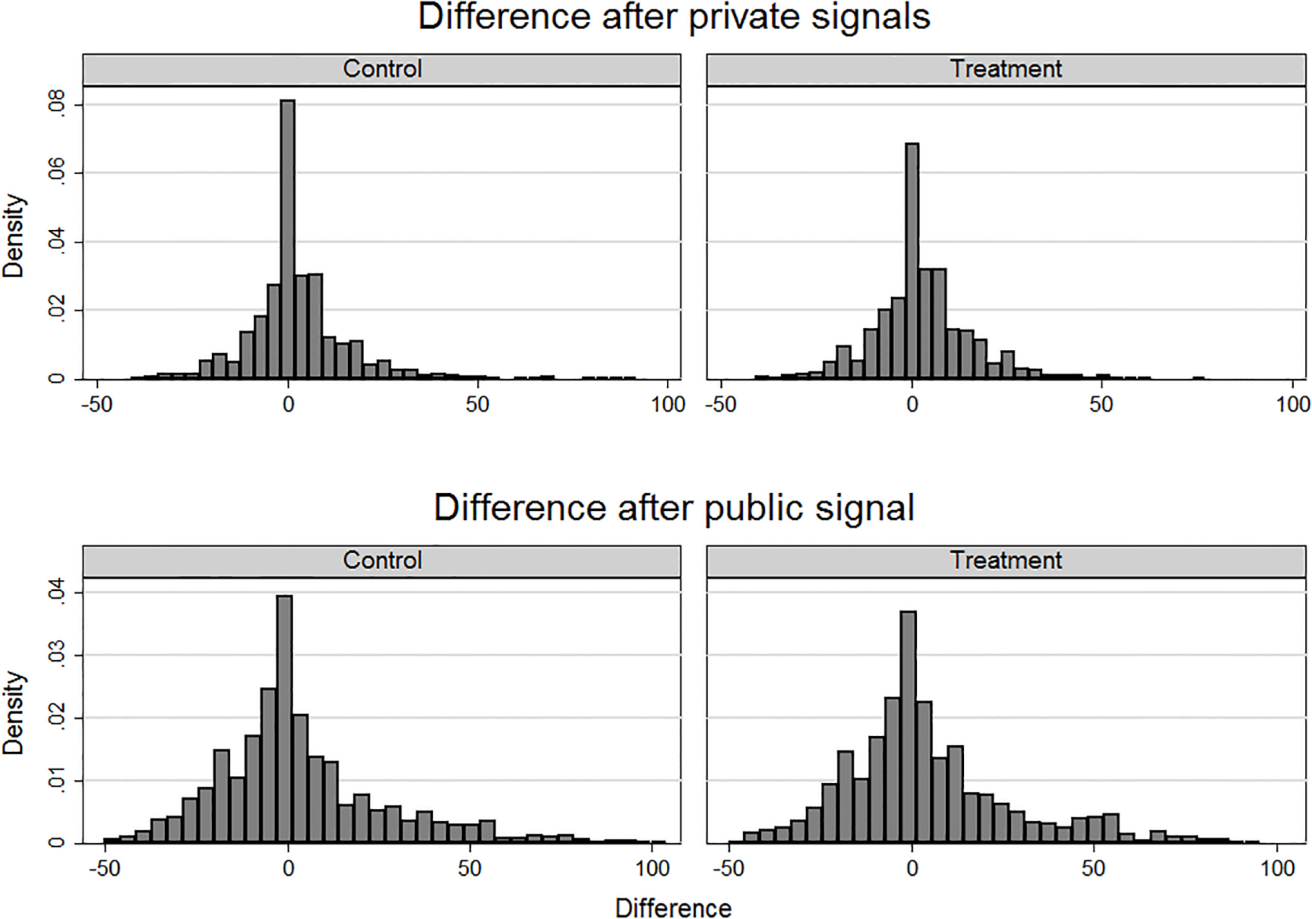
Histogram of variable *Difference*, by the type of signal and treatment status.

When examining the density of the variable *Difference*, it reveals that the subjects reacted rather rationally to the signals they had received since the distribution is centered on the mean of zero and is not skewed to either of the sides. When divided into the two types of signals after treatment (Fig 6), qualitatively no difference emerges.

### 3.4. Regression analysis

Since I observe the decisions of both groups before and after the treatment manipulation, I analyze the level differences using the difference-in-differences approach, even though accounting for the behavior before the treatment intervention may be considered as an overly conservative approach. Both treatment and control groups faced identical structure of signals in a given round, so on average they faced signals of the same strength and in the same order. The analysis is thus first comparing only the average reactions of the treatment and the control groups across the two periods, while I analyze the role of time dimension together with other robustness checks in more detail in the latter sections. To account for the potential correlation between decisions made by a single individual, I cluster standard errors in the regressions on an individual level.

Table 2 presents the main results of the regression analysis. There I regress the variable *Decision* on *Treatment* dummy, the dummy indicating decisions made after the treatment procedure *Round after stress*, and their interaction *Treatment X Round after TSST*, which constitutes the standard difference-in-differences setting. To investigate the differential reaction to private and public signals, I further include the following set of variables: *Public* is a dummy indicating the public signal, *Public X Treatment* indicates decisions of subjects in the treatment group when reacting to public signals, *Public X Round after TSST* indicates the decisions made in reaction to public signals after the treatment procedure, and the triple interaction *Public X Round after TSST X Treatment* is the coefficient of interest as it indicates how different before and after the treatment intervention a reaction to a public compared to private signal between the treatment and control group. After running the baseline model, I add the session fixed effects to account for the unobserved heterogeneity across sessions (column 2). In column 3 I add the individual observable characteristics, i.e. the Big-Five personality traits, age, the trait conformity, and a dummy indicating female. In column 4 I finally add a set of variables that should reveal a differential reaction of treatment group after the treatment intervention to signals of the same strength. The coefficient of interest is again the triple interaction *True X Round after TSST X Treatment*.

**Table 2:** Regression analysis, dependent variable *Decision*.

	(1)	(2)	(3)	(4)
Dependent variable	Decision			
Treatment	0.535	0.535	1.270	0.979
	(1.323)	(1.256)	(1.222)	(1.289)
Round after TSST	1.105	1.105	1.105	-2.269**
	(0.733)	(0.733)	(0.734)	(0.958)
Treatment X Round after TSST	-0.0460	-0.0460	-0.0460	-0.199
	(0.959)	(0.959)	(0.959)	(1.083)
Public	2.729***	2.729***	2.729***	2.095***
	(0.653)	(0.654)	(0.654)	(0.642)
Public X Treatment	0.320	0.320	0.320	0.0160
	(0.936)	(0.936)	(0.937)	(0.920)
Public X Round after TSST	0.749	0.749	0.749	-0.00931
	(0.720)	(0.721)	(0.721)	(0.709)
Public X Round after TSST X Treatment	-0.0150	-0.0150	-0.0150	0.697
	(0.945)	(0.946)	(0.946)	(0.911)
True				0.369***
				(0.0289)
True X Treatment				0.0238
				(0.0376)
True X Round after TSST				0.142***
				(0.0258)
True X Round after TSST X Treatment				-0.0256
				(0.0315)
Constant	20.92***	19.58***	21.81**	14.25
	(0.933)	(2.576)	(9.486)	(9.658)
Observations	9,240	9,240	9,240	9,240
R-squared	0.011	0.024	0.043	0.379
Session FE	NO	YES	YES	YES
Controls	NO	NO	YES	YES
F	14.52	7.943	6.116	41.76

*Notes*: Dependent variable *Decision*. Robust standard errors clustered on the individual level in the parentheses.

*** p<0.01.

** p<0.05.

* p<0.1.

The results generally show a robust null effect. First note that the coefficient of the variable *Treatment X Round after stress* is not different from zero in any of the specifications (all p’s > 0.36), which informs us that the general weight of either type of a signal was not different for both treatment and the control groups after the TSST-G procedure. Its largest magnitude -0.199 corresponds to about 0.01 SD. Next the coefficient of the triple interaction *Public X Round after TSST X Treatment* is also not significantly different from zero which implies that the weight of public signals after the TSST-G procedure was not different in the treatment and the control groups. The significant and positive coefficient of the indicator variable *Public* informs us that the signal from public sources followed by about 2.7 points more than the private signal, which is actually inconsistent with the literature [99,100] where the typical finding is that people tend to rely more on private than on public information. The significance of the control variable *True* shows that subjects used the information contained in the signals, and for each percentage point in the probability predicted by the Bayes formula they moved the slider on average by 0.37 points in the correct direction. The triple interaction term *True X Round after TSST X Treatment* is insignificant and small in magnitude, which suggests that the difference between the treatment and control group in the how they react to the strength of signals did not change compared to the situation before the treatment intervention. Further note the insignificance of the term *Treatment* reveals that there are no systematic differences between the two groups before the treatment intervention and the term *Round after stress* shows no difference between the decisions made before the treatment procedure and after the procedure in the control group. From the control variables, coefficient of *Age* is significant and positive, which means that older subjects stated probability after a private signal larger than the younger ones.

For a better clarity I present in the SOM the analysis re-run only on decisions made after the private (Table A4 in S1 File) and only public (Table A5 in S1 File) signals, where I also show the coefficients of the control variables. Personality traits are insignificant with the exception of Neuroticism (and for public signals, also Agreeableness) which is significant on a 1% level and negative, which means that subjects that are more neurotic and emotionally unstable stated a probability smaller than other subjects. Also, coefficient of Age is significant and positive, which informs us that generally older subjects tend to state higher stated probability. In addition to the analysis above I also test a gender-specific effect of the treatment by adding a set of dummy variables *Female*, *Treatment X Female* and *Treatment X Female X Round after stress* (column 3 in both tables). The triple interaction is not significant in any of the two tables, and the coefficient of *Treatment X Round after stress* also does not change. Finally I test for an order effect (column 6) when I restrict the observations to only the first private or public signals each subject received in a given round to conclude that there was no difference. Even then the coefficient of Treatment *Round after stress does not change its size or significance.

### 3.5. Precision of estimates

Another potential effect of stress was hypothesized to appear in the precision of individual estimates relative to the value predicted by the Bayes theorem. Using again the same difference-in-differences approach I regress the variable *Difference* in an absolute value on the sets of variables as in the preceding sections, only separately for private and public signals. In Table 3 I show that for both types of the signal, I cannot reject that the precision of estimates is the same for both treatment and control groups since the coefficient of Treatment *Round after stress is not significantly different from zero in all specifications. The coefficient *Round after stress* is significant and negative for the decisions made after the public signal which shows that both treatment and control groups improved the precision of the estimates after the TSST-G procedure. The addition of personality controls (columns 3 and 7) does not change anything. When investigating the role of the size of the Bayesian prediction by adding variable *True* and the appropriate set of interactions for the diff-in-diff-in-diff, we observe the triple interaction marginally significant (p = 0.078) and positive for the private signals (column 4) which means that the precision of subjects in treatment group was relatively to the control group a little worse after the treatment intervention. For the public signals this relationship (column 8) however does not hold.

**Table 3:** Regression analysis, dependent variable *Difference* in absolute value.

	(1)	(2)	(3)	(4)	(5)	(6)	(7)	(8)
	Difference in absolute value							
Signal type:	Private				Public			
Treatment	0.499	0.499	0.205	0.982	-0.302	-0.302	-0.336	0.398
	(0.979)	(0.947)	(0.960)	(1.944)	(1.054)	(1.033)	(1.024)	(1.373)
Round after TSST	-0.0728	-0.0728	-0.0728	0.259	-2.667***	-2.667***	-2.667***	-1.905**
	(0.538)	(0.539)	(0.539)	(1.345)	(0.617)	(0.618)	(0.619)	(0.944)
Treatment X Round after TSST	-0.110	-0.110	-0.110	-2.044	0.741	0.741	0.741	0.328
	(0.669)	(0.669)	(0.670)	(1.573)	(0.822)	(0.823)	(0.824)	(1.184)
True				-0.115*				-0.339***
				(0.0624)				(0.0363)
True X Treatment				-0.0471				-0.0338
				(0.0752)				(0.0484)
True X Round after TSST				-0.00730				0.00606
				(0.0523)				(0.0299)
True X Round after TSST X Treatment				0.110*				0.0206
				(0.0624)				(0.0373)
Constant	8.809***	12.23***	11.41	13.81*	17.81***	20.37***	29.78***	36.27***
	(0.706)	(2.241)	(8.092)	(7.850)	(0.808)	(2.238)	(8.651)	(6.434)
Observations	5,320	5,320	5,320	5,320	3,920	3,920	3,920	3,920
R-squared	0.000	0.025	0.046	0.085	0.005	0.011	0.019	0.299
Session FE	NO	YES	YES	YES	NO	YES	YES	YES
Controls	NO	NO	YES	YES	NO	NO	YES	YES
F	0.130	1.334	1.992	2.397	10.47	3.807	3.644	19.06

*Notes*: Dependent variable: *Difference* in absolute value. Robust standard errors clustered at the individual level in parentheses.

*** p<0.01.

** p<0.05.

* p<0.1.

### 3.6. Robustness checks

To investigate whether there were any different treatment effects with respect to the reactivity to stress, I also perform the difference-in-differences comparison of the cortisol responders and non-responders, where I define that a subject is a cortisol responder as 1 if cortisol increased by at least 1.5 nmol/l or 15.5% between the baseline (sample 1) and the higher of the two samples taken after the treatment procedure [101] (see Tables A6 and A7 in S1 File for the distribution of the responders in the treatment and control groups). I re-run the regression models in Tables 2 and 3 to conclude that the results are robust against this change of specification, which would reveal correlations between being stressed and a change in behavior (results in Table A8 in S1 File).

I further checked for a correlation between the cortisol increase and the variables of interest (*Decision* and *Difference* in absolute value) for the treatment group only to see that the interaction of the cortisol increase *Responder* with *Round after stress* is not significant. I then substitute the increase in cortisol with the change in heart-rate during the procedure, change in mood in the good-bad and the calm-nervous dimensions to see again no effect on any of the main variables of interest. Also when I investigate treatment-specific reaction with respect to observable personal controls and interact them with *Treatment*, results hold unchanged and none of the interactions is significant (Results available upon request).

Next, to account better for the time-dimension of decisions I re-run the analysis for the main specifications using random-effects panel data estimation with the unit-level being the individual and the time-dimension being the order of the decision made by an individual in an experiment; and still cluster the standard errors on the individual level. Table A9 in S1 File shows that again, no qualitative difference in the results emerges. Next I perform two more exercises where I take the main specifications and add two variables that represent the time dimension, the order number of the round and the order number of a single individual decision, and their interactions with Treatment. For a better clarity I restrict the analysis to the periods after the treatment intervention (proper randomization allows for this move) and in Tables A10 and A11 in S1 File I show that the coefficients of variables of interest remain unaffected.

As mentioned earlier, an important potential confounding factor is a treatment-specific change in risk-preferences, especially when the quadratic scoring rule is incentive compatible only under risk-neutrality. First note that the QSR procedure was constant across treatments and due to the random distribution of subjects into treatment and control we can reasonably assume a similar a-priori distribution of risk-preference profiles in both groups. Second, the increase in risk-aversion would probably induce more conservative updating behavior and subjects would generally keep the slider closer to the midpoint (hedge more), which is however not what is observed in the data. As I observe a null result, a potential change in risk-preferences may have offset the change in updating behavior. This effect may be revealed by controlling for the risk-preferences in the above-carried regressions, even though such move may introduce an endogeneity bias. Recall that risk-preference profiles were measured by the incentivized risk-elicitation task. For 136 subjects we elicit a meaningful certainty equivalent, as four subjects indicated inconsistent choices. In Table A12 in S1 File three key regression specifications are re-run with the addition of the Certainty equivalent. The results allow us to conclude that the main coefficient of interest of the diff-in-diff interaction does not change its magnitude or insignificance, and the role of risk-preference change seems irrelevant.

### 3.7. Variance analysis

To further check for possible differences in treatment effects I also conduct the analysis of the equality of variance between the treatment and control groups. The variance ratio test revealed no differences between the two groups in the variable *Decision*; either before (p = 0.94) or after (p = 0.77) the TSST-G procedure. The Kolmogorov-Smirnov test for the equality of distribution functions also showed no differences (corrected p-values: before, p = 0.18; after, p = 0.3).

When carried out for the variable *Difference* in absolute value, the Kolmogorov-Smirnov test revealed no difference before (corrected p = 0.36), but after (p = 0.013) the TSST-G procedure. When examined with the robust test for the equality of variances, the differences disappear (p > 0.37 and above). I implemented this test in Stata 12 in the command *robvar*, which uses the Levene’s robust statistic. Since the distribution is highly skewed (p<0.001), a standard test of the equality of variances would deliver biased results.

## 4. Discussion

The subjects in the treatment group were indeed physiologically and psychologically stressed: The success of the manipulation is demonstrated using two physiological (salivary cortisol levels and heart-rate) and one psychological (Multidimensional Mood Questionnaire scores, [102]) measures. I focus on salivary cortisol since it has been established as a reliable biomarker of the HPA activation during acute stress [103,104]. While not different between the groups during the baseline measurement, cortisol levels slightly decreased in the control group during the control procedure while they almost doubled in the treatment group. Heart-rate was on average 10 beats per minute higher for the treatment group than for the control group. The mood scores showed treatment group felt significantly worse and more nervous than the control group. Overall, these measures show that the magnitude of stress induced in subjects was in a comparable manner as in the related literature [66,70].

The main behavioral result stemming from an experiment with 140 subjects which is comparable or larger than what has typically been used in the literature [6,86,105], is that stress has robust null effect on social learning, i.e. the weight which both the public and private signals are given in the decisions of subjects is not statistically different between the treatment (stress) and the control (non-stress) groups. The precision of the individual point estimates also does not systematically differ, although in one specification the difference in precision after private signals between the treatment and control groups in the reaction to the signal strength (the Bayesian prediction) was larger after the treatment manipulation, but it was marginally significant (p = 0.078) and small in magnitude. This could be interpreted such that with stronger signals the treatment group made larger deviations from the optimal moves.

The general null result is robust to the addition of various control variables, including psychological measure of conformity and personality traits. Moreover, the data replicates common findings in the literature concerning the updating behavior as I observe common behavioral regularities, such as the clustering of probability estimates on multiples of five, conservativeness and an updating behavior generally consistent with the Bayes theorem [49,106,107]. I also find that public signal is generally taken into account more during updating and older subjects are more conservative than younger ones. From the personality traits only Neuroticism is consistently significant across specifications and negative in sign, which suggests that subjects high in this trait are also generally more conservative.

In the meta-analysis of information-cascade studies, Weizsäcker [99] finds that private signals are taken more seriously than public signals in a 2:1 ratio, while I observe an opposite finding. This could be due to several reasons. First, subjects could have really understood the signal as something more informative, which is however unlikely to have happened. Second, our calculation of the Bayesian benchmark included an assumption that in case of 50/50 a random color would be chosen, which could have been disregarded by subjects. Third, the mere fact that they were shown later in the sequence of all signals in a given round may have caused that they were taken more seriously than they should have been as the uncertainty over which bag had been selected was relatively small.

The absence of finding the hypothesized relationships may be due to several reasons. Either the design was too noisy and unable to correctly identify the proposed relationship, or the relationship is smaller than could be found with the statistical power in this design, or the relationship is indeed not there, or two opposing stress effects may have cancelled each other out [105]. I cannot rule out the last possibility since for example the change in preferences and change in expectations may have yielded opposing effects: subjects may have been more likely to take into account the information from the public signal due to a change in their preferences, but at the same time, since they knew others were also under stress and may have reacted to the new situations even more, they may have discounted their beliefs about the real value of public signal.

To check the statistical power of my design I first used G*POWER 3.1 [108] to assess the *post-hoc* statistical power of the t-tests in the multiple regression with four to thirteen predictors. The analysis revealed that with the sample size of 140 subjects and the power of 0.8, my design was able to detect a small to medium effect size of *f*^*2*^ = 0.044 to 0.06, depending on the specification (see Section C in S1 File in SOM for the output from G*POWER). The conventional values for the effect size in this test are for small effect *f*^*2*^ = 0.02, medium effect *f*^*2*^ = 0.15 and for large effect *f*^*2*^ = 0.35 [108,109]. Next I calculated the minimal detectable effect size as suggested by Haushofer & Shapiro [110]: with the power of the test of 0.8 and the significance level of 0.05, the *post-hoc* minimal detectable effect size is given by

$$MDE=(t_{1-\kappa }+t_{\left(\frac{\alpha }{2}\right)})\times \frac{\sigma }{\sqrt{NP(1-P)}}$$

where *t*_1−*k*_ is the t-statistic value required for obtaining 80% power, *t*_(*α*/2)_ is the critical value required to achieve the 5% significance level, *P* is the fraction of the sample that was treated and the remaining fraction is the standard error of the coefficient of interest. With *t*_1−*k*_ = 0.84, *t*_(*α*/2)_ = 1.96 and *P* = 0.5 is thus $MDE=2.8\times SE(\hat{\beta })$, i.e. a simple multiple of the standard error of estimated coefficient of a standardized variable. This approach yields size of the effect between 0.14 and 0.16 SD (Table A13 in S1 File), which corresponds to a rather small effect size that was possible to detect with this design. Therefore it seems unlikely that the null result is due to insufficient power of the experimental design.

If stress causes changes in risk preferences, I should observe a systematic difference between the treatment and control groups after the treatment manipulation; i.e. the coefficient of the variable Treatment*Round after stress should be significantly different from zero. However, I do not observe any such effect. Either the effect is too small to be identified in the regressions, or it is already captured in some of the control variables, or it is offset by a combination of other factors, such as differences in beliefs about others and about the riskiness of the signals. Another potential reason may be hidden in the opposing effects of an increased reward responsiveness under stress [111] and increased risk aversion that cancel each other out. When controlling for the certainty equivalent in the regressions I show that this is unlikely to explain the null effects.

Another possible explanation for the null result could arise despite all my efforts to avoid it by implementing the design feature of constantly changing the setting of the parameters of the problem, the updating process in the task could have already been mastered in the rounds before the stress procedure. In such a case, the decision making process would be operated by the fast automatic, Type-I processes rather than by the slower Type-II, rational processes. Indeed, in a recent study Bayesian updating has been found to be governed rather by automatic processes than by rational thought [52]. If a stress reaction affects the higher-cognitive functions operated in the prefrontal cortex as is generally suggested in the literature [63,74,76], but does not impair habitual automatic behavior, my design was not able to capture the effects of stress on social learning initiated in the higher-order cognitive structures.

In the related literature investigating the effects of stress on economic decision making, the results in many domains are generally mixed and this is by far not the first study to find null effects. No effects of acute stress were found on inter-temporal discounting [105], non-social risk-taking [6], and ambiguity aversion [112]. However, other studies found positive results, e.g. in the realm of risk-preferences, willingness to compete or social preferences [6,9,55,84,96,113,114]. Moreover, the effects of stress can basically vary by the stressor used [115,116] and the timing when the behavioral task was administered [117], which may also explain why I observe different results than Buckert, Oechsler et al. [13].

## 5. Conclusion

As one of the first studies of this type in the literature, using an efficient stressor and a standard Bayesian updating task I provide evidence that there is no effect of acute stress on social learning. Using salivary cortisol levels, heart rate and changes in mood I demonstrate that unlike participants in the control group, participants in the treatment group were under considerable levels of stress. The use of information in the process of Bayesian updating as well as the precision of the subjective estimates does not differ for the participants who underwent a stress-inducing treatment procedure and the control participants, and this is true for both private and public signals they received. I further conduct several robustness checks to prove that this null result is not due to different reactions of stressed and non-stressed subjects in terms of the cortisol increase, different gender reactions to stress, differences in personality, and due to subject-specific and session-specific effects. My results thus suggest that despite the existing literature on the effects of acute stress on decision making [5], individual-level social learning behavior is not affected by mild acute psycho-social stress, though I cannot conclude the existence of effects of a more severe or a different type of stress [115].

If we assume that the daily routine behavior of decision makers, e.g. professional traders, is more a habitual than a higher cognitive activity, the results of this study imply that the observed real-world phenomena when people engage in social learning behavior in stressful situations, such as bank-runs, herding in financial markets during increased volatility and panic in general, as well as the results of the related studies [12,13], occur due to changes in a different dimension of human behavior than social learning and information updating, with the likely candidates being risk preferences, beliefs about the behavior of others and the general adaptation to a new environment. The real underlying reasons of these phenomena should thus be investigated in the future research.

## Supporting information

S1 File Additional results. This file contains additional results referred to from the main text. It has sections labeled A to D. Section A contains 13 tables labeled Table A1 to Table A13; section B contains text discussing the role of risk preferences in detail; section C contains output from a program G-Power to support calculations made in the main text, and section D contains a detailed description of the calculation procedure of the variable *True*.
(PDF)

S2 File TSST-G protocol. This file contains a detailed description of the treatment procedure, including a scheme of the room, the script and instructions for both treatment and control groups, and the text used for the debriefing of the treatment group.
(PDF)

S3 File Experimental instructions. This file contains the instructions that were given to subjects about the task measuring the updating behavior that is described in the main text in section 3.1.
(PDF)

S4 File Control questions. This file includes the exact wording of the control questions that were checking the understanding of subjects to the rules of the main task.
(PDF)

S5 File Datasets and do-files. This compressed file includes the data as gathered in the experiment and do-files that produced the tables in the text. The datasets are in the Stata 13 format and are two for the sake of convenience of computation. The datafile S5_data_personality.dta and its associated do-file S5_do_personality.do were used for the production of the Tables A1 and A2 (manipulation and randomization checks). Datafile S5_data_main.dta and associated do-file S5_do_main.do were used for the production of the rest of the tables.
(ZIP)
