# Supplementary material for: Social learning under acute stress
Source: PLoS One. 2018 Aug 22;13(8):e0202335. doi: 10.1371/journal.pone.0202335 (PMC6104985; doi:10.1371/journal.pone.0202335)
Supplement: S1 File — This file contains additional results referred to from the main text. It has sections labeled A to D. Section A contains 13 tables labeled Table A1 to Table A13; section B contains text discussing the role of risk preferences in detail; section C contains output from a program G-Power to support calculations made in the main text, and section D contains a detailed description of the calculation procedure of the variable True. (PDF) [file pone.0202335.s001.pdf]

Supporting online material - file 1

Section A

Table A1: Randomization check

		Total	Control	Treatment	p-value
Gender	Male (%)	52.1	52.9	51.4	0.87
	Female (%)	47.9	47.1	48.6	
	Count	140	70	70	
Age	Mean	22.3	22.6	22.1	0.51
	SD	2.4	2.8	1.8	
	Valid N	140	70	70	
Education	Elementary (%)	0.0	0.0	0.0	
	High school (%)	55.7	60.0	51.4	0.35
	University (%)	44.3	40.0	48.6	
	Count	140	70	70	
Openness to experience	Mean	36.8	37.1	36.5	0.26
	SD	5.3	5.6	5.0	
	Valid N	140	70	70	
Conscientiousness	Mean	33.6	34.0	33.2	0.38
	SD	6.2	6.6	5.9	
	Valid N	140	70	70	
Extraversion	Mean	33.2	32.1	34.3	0.08
	SD	7.7	7.9	7.4	
	Valid N	140	70	70	
Agreeableness	Mean	36.2	35.9	36.5	0.33
	SD	5.8	5.1	6.4	
	Valid N	140	70	70	
Neuroticism	Mean	30.2	29.0	31.4	0.08
	SD	7.8	8.1	7.4	
	Valid N	140	70	70	
Conformity	Mean	28.6	28.7	28.5	0.92
	SD	5.7	6.0	5.4	
	Valid N	140.0	70.0	70.0	

Note: p-values from the two-sample Wilcoxon rank-sum test.

Table A2: Manipulation check

		Control	Treatment	p-value<br>z-value<br>Cohens d	Male		Female	
					Control	Treatment	Control	Treatment
Cortisol 1 - Before treatment	Mean	7.76	8.21	0.83	6.81	8.45	8.80	7.95
	SD	3.89	4.39	-0.21	4.11	4.59	3.41	4.22
	Valid N	69	70	-0.1	36	36	33	34
Cortisol 2 - After treatment	Mean	6.97	16.79	0.00	7.02	19.27	6.92	14.17
	SD	4.16	11.96	-6.22	5.20	12.10	2.62	11.39
	Valid N	70	70	-1.1	37	36	33	34
Cortisol 3 - Before risk-task	Mean	7.07	16.13	0.00	7.07	17.99	7.06	14.09
	SD	4.24	11.64	-6.05	5.13	11.00	3.06	12.15
	Valid N	69	69	-1.04	36	36	33	33
Heart rate - Before treatment	Mean	80.68	82.43	0.44	79.91	78.89	81.63	86.09
	SD	12.77	12.53	-0.77	12.89	12.40	12.79	11.76
	Valid N	65	65	-0.14	36	33	29	32
Heart rate - During treatment	Mean	92.92	100.99	0.07	90.88	96.31	95.74	105.97
	SD	20.41	19.25	-1.84	14.95	16.08	26.25	21.27
	Valid N	62	64	-0.41	36	33	26	31
Heart rate - after treatment	Mean	79.87	81.70	0.32	76.22	79.06	84.43	84.41
	SD	16.01	12.88	-0.99	12.98	12.64	18.37	12.74
	Valid N	63	65	-0.12	35	33	28	32
MDM Good-Bad 1 - before Treatment	Mean	22.89	23.36	0.29	23.41	23.19	22.30	23.53
	SD	4.40	4.45	-1.05	4.75	4.70	3.97	4.23
	Valid N	70	70	-0.11	37	36	33	34
MDM Good-Bad 2 - after Treatment	Mean	21.31	17.79	0.00	22.00	18.31	20.55	17.24
	SD	4.57	5.71	3.60	4.76	5.83	4.30	5.62
	Valid N	70	70	0.68	37	36	33	34
MDM Awake-Tired 1 - before Treatment	Mean	20.41	20.44	0.83	21.14	21.60	19.61	19.24
	SD	5.53	4.83	-0.22	5.53	5.10	5.50	4.29
	Valid N	70	69	-0.001	37	35	33	34
MDM Awake-Tired 2 - after Treatment	Mean	18.83	19.91	0.11	19.54	20.81	18.00	18.97
	SD	5.15	5.36	-1.59	4.29	5.04	5.95	5.61
	Valid N	69	70	-0.1	37	36	32	34
MDM Calm-Nervous 1 - before Treatment	Mean	21.79	22.91	0.23	21.97	22.47	21.58	23.39
	SD	4.77	4.49	-1.20	4.91	4.10	4.68	4.89
	Valid N	70	69	-0.24	37	36	33	33
MDM Calm-Nervous 2 - after Treatment	Mean	20.10	17.22	0.00	20.87	18.00	19.24	16.36
	SD	4.70	4.85	3.44	4.76	4.52	4.54	5.13
	Valid N	70	69	0.61	37	36	33	33

Note: p-values from the two-sample Wilcoxon rank-sum test.

Table A3: Descriptive statistics of main independent variables.

		Control	Treatment	Control	Treatment
		(1)	(2)	(3)	(4)
		Private signals		*Public signals*	
VARIABLE		*Before TSST procedure*			
Decision	Mean	20.9	21.5	23.6	24.5
	SD	15.8	15.7	16.8	16.9
Difference	Mean	3.1	4.2	4.1	4.9
	SD	13.5	12.9	25.2	24.6
Difference abs	Mean	8.8	9.3	17.8	17.5
	SD	10.7	9.9	18.2	17.9
		After TSST procedure			
Decision	Mean	22.0	22.5	25.5	26.3
	SD	16.8	16.7	18.1	18.0
Difference	Mean	2.5	2.3	3.3	4.1
	SD	13.5	12.9	21.6	21.9
Difference abs	Mean	8.7	9.1	15.1	15.6
	SD	10.6	9.3	15.7	15.9

Table A4: Regression analysis, dependent variable *Decision*, only decisions after Private signals.

	(1)	(2)	(3)	(4)	(5)	(6)
				**Private signals**		
Dependent variable				Decision		
Treatment	0.535	0.535	-0.384	1.309	1.077	2.176
	(1.323)	(1.251)	(1.458)	(1.207)	(1.732)	(1.767)
Round after TSST	1.105	1.105	1.105	1.105	-0.482	-0.731
	(0.733)	(0.733)	(0.734)	(0.734)	(1.286)	(1.374)
Treatment X Round after TSST	-0.0460	-0.0460	0.0181	-0.0460	-1.264	-1.770
	(0.958)	(0.959)	(1.199)	(0.960)	(1.489)	(1.801)
Female			-2.424	-1.697	-0.329	1.288
			(1.719)	(1.368)	(1.156)	(1.301)
Age				0.506**	0.577***	0.485**
				(0.215)	(0.213)	(0.219)
Openness to experience				-0.0525	-0.137	-0.131
				(0.135)	(0.128)	(0.133)
Conscientiousness				0.0753	0.0926	0.151*
				(0.0888)	(0.0851)	(0.0898)
Extraversion				0.0838	0.123	0.0974
				(0.0937)	(0.0835)	(0.0891)
Agreeableness				-0.159	-0.149	-0.153
				(0.106)	(0.100)	(0.110)
Neuroticism				-0.239***	-0.208***	-0.207***
				(0.0787)	(0.0696)	(0.0703)
Conformity				-0.0462	-0.0572	-0.0310
				(0.119)	(0.0993)	(0.100)
True					0.619***	0.637***
					(0.0551)	(0.0616)
True X Treatment					0.0243	-0.0280
					(0.0724)	(0.0986)
True X Round after TSST					0.0293	-0.0145
					(0.0446)	(0.0628)
True X Treatment X Round after TSST					0.0147	0.0403
					(0.0532)	(0.0875)
Female X Treatment			1.963			
			(2.587)			
Female X Treatment X Round after TSST			-0.132			
			(1.229)			
Constant	20.92***	19.60***	20.68***	20.02**	7.271	4.375
	(0.933)	(2.444)	(2.461)	(9.172)	(9.230)	(8.735)
Observations	5,320	5,320	5,320	5,320	5,320	1,820
R-squared	0.001	0.017	0.020	0.034	0.525	0.400
Session FE	NO	YES	YES	YES	YES	YES
F	1.810	1.803	1.588	2.023	32.47	22.39

Notes: Dependent variable *Decision*. Robust standard errors clustered on the individual level in the parentheses. Column 6 restricts the observations to the first private signal in a given round. *** p<0.01, ** p<0.05, * p<0.1.

Table A5: Regression analysis, dependent variable *Decision*, only decisions after Public signals.

	(1)	(2)	(3)	(4)	(5)	(6)
				**Public signals**		
Dependent variable				Decision		
Treatment	0.855	0.855	-0.162	1.536	1.053	1.634
	(1.519)	(1.483)	(1.858)	(1.442)	(1.576)	(1.607)
Round after TSST	1.854*	1.854*	1.854*	1.854*	-1.706	-3.227**
	(0.983)	(0.984)	(0.984)	(0.985)	(1.071)	(1.335)
Treatment X Round after TSST	-0.0610	-0.0610	0.199	-0.0610	0.779	0.292
	(1.218)	(1.219)	(1.367)	(1.220)	(1.307)	(1.681)
Female			-3.859**	-2.683*	-1.584	-1.205
			(1.817)	(1.530)	(1.395)	(1.511)
Openness to experience				-0.0553	-0.101	-0.112
				(0.150)	(0.156)	(0.168)
Conscientiousness				-0.00418	-0.00336	0.0268
				(0.0986)	(0.0992)	(0.111)
Extraversion				0.132	0.143	0.155
				(0.105)	(0.102)	(0.108)
Agreeableness				-0.271**	-0.275**	-0.280**
				(0.134)	(0.134)	(0.139)
Neuroticism				-0.231**	-0.225***	-0.217**
				(0.0927)	(0.0856)	(0.0925)
Conformity				-0.0286	-0.0611	-0.146
				(0.139)	(0.128)	(0.132)
Age				0.536**	0.620**	0.595**
				(0.243)	(0.253)	(0.265)
True					0.263***	0.162***
					(0.0245)	(0.0320)
True X Treatment					0.0217	-0.0501
					(0.0323)	(0.0450)
True X Round after TSST					0.129***	0.245***
					(0.0237)	(0.0411)
True X Round after TSST X Treatment					-0.0386	0.0270
					(0.0311)	(0.0559)
Female X Treatment			2.206			
			(3.029)			
Female X Treatment X Round after TSST			-0.535			
			(1.443)			
Constant	23.65***	22.27***	23.95***	26.96**	21.33*	25.66**
	(0.974)	(2.972)	(3.046)	(10.47)	(10.88)	(11.19)
Observations	3,920	3,920	3,920	3,920	3,920	1,820
R-squared	0.003	0.015	0.022	0.039	0.280	0.248
Session FE	NO	YES	YES	YES	YES	YES
F	3.479	1.855	1.671	2.237	38.49	30.42

Notes: Dependent variable *Decision*. Robust standard errors clustered on the individual level in the parentheses. Column 6 restricts the observations to the first public signal in a given round.*** p<0.01, ** p<0.05, * p<0.1

Table A6: Classification of subjects whether they reacted to the treatment procedure according the increase in cortisol above 1.5 nmol/l above the baseline.

		Responder - Cortisol increase above 1.5 nmol/l		
		No	Yes	Total
Group:	Control	56	12	68
	Treatment	18	52	70
	Total	74	64	138

Table A7: Classification of subjects whether they reacted to the treatment procedure according the increase in cortisol above 15.5 % above the baseline.

		Responder - Cortisol increase above 15.5%		
		No	Yes	Total
Group:	Control	51	17	68
	Treatment	16	54	70
	Total	67	71	138

Table A8: Regression analysis - correlations.

	(1)	(2)	(3)	(4)	(5)	(6)	(7)	(8)
Dependent variable		Decision				Difference in absolute value		
Responder	1.173	1.361	1.017	1.168	0.482	0.427	0.935	0.914
	(1.467)	(1.434)	(1.437)	(1.386)	(0.958)	(0.926)	(0.917)	(0.845)
Round after TSST	1.485**	1.488**	1.466**	-1.905**	-0.0698	-0.0699	-0.0585	-0.617
	(0.687)	(0.688)	(0.689)	(0.751)	(0.505)	(0.505)	(0.505)	(0.688)
Responder X Round after TSST	-0.848	-0.854	-0.811	-0.968	-0.125	-0.125	-0.147	-0.228
	(1.043)	(1.043)	(1.045)	(0.914)	(0.610)	(0.611)	(0.611)	(0.607)
Public	2.787***	2.840***	2.425***	2.031**	9.010***	9.008***	9.222***	9.469***
	(0.938)	(0.935)	(0.911)	(0.860)	(0.706)	(0.681)	(0.680)	(0.676)
Public X Treatment	0.171	0.0667	0.885	0.120	-0.714	-0.710	-1.133	-0.431
	(1.670)	(1.659)	(1.608)	(1.387)	(1.075)	(1.038)	(1.003)	(1.063)
Public X Round after TSST	0.395	0.393	0.405	-0.234	-2.602***	-2.602***	-2.608***	-2.288***
	(0.891)	(0.892)	(0.892)	(0.785)	(0.627)	(0.628)	(0.627)	(0.615)
Public X Round after TSST X Treatment	0.567	0.570	0.547	1.021	0.842	0.842	0.853	0.0777
	(1.302)	(1.303)	(1.304)	(1.118)	(0.841)	(0.842)	(0.841)	(0.908)
True				0.364***				-0.276***
				(0.0274)				(0.0341)
True X Treatment				0.0301				-0.0319
				(0.0347)				(0.0366)
True X Round after TSST				0.141***				0.0437*
				(0.0222)				(0.0256)
True X Round after TSST X Treatment				-0.0204				0.0399
				(0.0267)				(0.0256)
Constant	20.64***	19.14***	21.08**	13.73	8.837***	11.86***	15.63**	20.83***
	(1.059)	(2.574)	(9.576)	(9.856)	(0.725)	(2.105)	(7.660)	(6.944)
Observations	9,108	9,108	9,108	9,108	9,108	9,108	9,108	9,108
R-squared	0.011	0.025	0.044	0.379	0.073	0.084	0.094	0.250
Session FE	NO	YES	YES	YES	NO	YES	YES	YES
Controls	NO	NO	YES	YES	NO	NO	YES	YES
F	13.12	7.350	5.642	41.75	85.56	42.81	33.05	47.68

Note: Responder defined as 1 if an individual showed an increase of cortisol higher than 1.5 nmol/l or 15.5% above the baseline, 0 otherwise. Dependent variables *Decision* (cols. 1-4) and *Difference* in absolute value (cols. 5-8). Robust standard errors clustered on the individual level in the parentheses. *** p<0.01, ** p<0.05, * p<0.1

Table A9: Random effects panel data estimation.

	(1)	(2)	(3)	(4)	(5)	(6)	(7)	(8)
Dependent variable		Decision				Difference in absolute value		
Treatment	0.535	0.535	1.270	1.368	0.499	0.499	0.315	0.965
	(1.323)	(1.256)	(1.222)	(1.263)	(0.979)	(0.946)	(0.954)	(1.366)
Round after TSST	1.105	1.105	1.105	-2.071**	-0.0728	-0.0728	-0.0728	-0.321
	(0.733)	(0.733)	(0.734)	(0.922)	(0.538)	(0.539)	(0.539)	(0.919)
Treatment X Round after TSST	-0.0460	-0.0460	-0.0460	-0.431	-0.110	-0.110	-0.110	-0.567
	(0.959)	(0.959)	(0.959)	(1.058)	(0.669)	(0.669)	(0.669)	(1.060)
Public	2.729***	2.729***	2.729***	2.079***	8.998***	8.998***	8.998***	9.429***
	(0.653)	(0.654)	(0.654)	(0.642)	(0.564)	(0.564)	(0.564)	(0.513)
Public X Treatment	0.320	0.320	0.320	0.0626	-0.801	-0.801	-0.801	-0.530
	(0.936)	(0.936)	(0.937)	(0.917)	(0.890)	(0.891)	(0.891)	(0.768)
Public X Round after TSST	0.749	0.749	0.749	0.0107	-2.594***	-2.594***	-2.594***	-2.434***
	(0.720)	(0.721)	(0.721)	(0.712)	(0.638)	(0.639)	(0.639)	(0.582)
Public X Round after TSST X Treatment	-0.0150	-0.0150	-0.0150	0.664	0.851	0.851	0.851	0.405
	(0.945)	(0.946)	(0.946)	(0.909)	(0.899)	(0.899)	(0.900)	(0.827)
True				0.379***				-0.251***
				(0.0276)				(0.0360)
True X Treatment				0.00157				-0.0433
				(0.0377)				(0.0462)
True X Round after TSST				0.131***				0.0339
				(0.0239)				(0.0292)
True X Round after TSST X Treatment				-0.0110				0.0444
				(0.0289)				(0.0342)
Constant	20.92***	19.58***	21.81**	14.06	8.809***	11.87***	15.38**	20.08***
	(0.933)	(2.576)	(9.486)	(9.615)	(0.706)	(2.150)	(7.806)	(7.095)
Observations	9,240	9,240	9,240	9,240	9,240	9,240	9,240	9,240
Number of units	140	140	140	140	140	140	140	140
Session FE	NO	YES	YES	YES	NO	YES	YES	YES
Controls	NO	NO	YES	YES	NO	NO	YES	YES

Note: Random effects panel data analysis with the unit of observation being an individual and time dimension being the serial order number of a decision across all rounds. Dependent variables *Decision* (cols. 1-4) and *Difference* in absolute value (cols. 5-8). Robust standard errors clustered on the individual level in the parentheses. *** p<0.01, ** p<0.05, * p<0.1

Table A10: Regression analysis – accounting for time by Round No. and Serial No, dependent variable *Decision*.

	(1)	(2)	(3)	(4)	(5)	(6)	(7)
VARIABLES				Decision			
Treatment	0.489	-1.572	-2.752	-2.157	-1.165	-2.098	-1.508
	(1.268)	(2.859)	(2.565)	(2.615)	(2.269)	(2.121)	(2.176)
Public	3.478***	3.441***	2.073***	2.070***	3.733***	2.282***	2.278***
	(0.475)	(0.474)	(0.507)	(0.507)	(0.485)	(0.524)	(0.524)
Public X Treatment	0.305	0.314	0.714	0.718	0.214	0.584	0.589
	(0.789)	(0.789)	(0.774)	(0.774)	(0.799)	(0.790)	(0.790)
Round No.		-0.679***	-0.451***	-0.451***			
		(0.158)	(0.135)	(0.136)			
Treatment X Round No.		0.177	0.241	0.240			
		(0.246)	(0.200)	(0.199)			
True			0.507***	0.508***		0.508***	0.510***
			(0.0345)	(0.0329)		(0.0345)	(0.0329)
True X Treatment			0.00246	0.000900		0.00207	0.000506
			(0.0407)	(0.0397)		(0.0408)	(0.0397)
Serial No.					-0.104***	-0.0761***	-0.0760***
					(0.0316)	(0.0269)	(0.0269)
Treatment X Serial No.					0.0367	0.0482	0.0481
					(0.0489)	(0.0394)	(0.0394)
Constant	20.44***	28.38***	16.09***	21.80*	25.11***	14.23***	19.93*
	(3.130)	(3.546)	(3.904)	(11.29)	(3.367)	(3.792)	(11.23)
Observations	5,740	5,740	5,740	5,740	5,740	5,740	5,740
R-squared	0.022	0.029	0.399	0.416	0.026	0.398	0.415
Session FE	YES	YES	YES	YES	YES	YES	YES
Controls	NO	NO	NO	YES	NO	NO	YES
F	8.949	9.305	54.74	46.49	8.646	54.49	46.08

Notes: Dependent variable *Decision*. Robust standard errors clustered on the individual level in the parentheses. *Round No* indicates the order number of a round in a session; *Serial No.* indicates the order number of a decision in a session.*** p<0.01, ** p<0.05, * p<0.1

Table A11: Regression analysis – accounting for time by Round No. and Serial No, dependent variable Difference in absolute value

	(1)	(2)	(3)	(4)	(5)	(6)	(7)
VARIABLES				Difference in absolute value			
Treatment	0.389	1.363	1.612	1.341	1.148	1.310	1.043
	(0.830)	(1.793)	(2.100)	(2.114)	(1.465)	(1.830)	(1.847)
Public	6.404***	6.400***	7.043***	7.036***	6.431***	7.110***	7.102***
	(0.462)	(0.461)	(0.419)	(0.421)	(0.472)	(0.430)	(0.432)
Public X Treatment	0.0493	0.0447	-0.165	-0.164	0.0907	-0.107	-0.106
	(0.670)	(0.669)	(0.599)	(0.601)	(0.685)	(0.616)	(0.619)
Round No.		-0.0800	-0.187**	-0.186**			
		(0.0926)	(0.0792)	(0.0796)			
Treatment X Round No.		-0.0834	-0.107	-0.107			
		(0.140)	(0.122)	(0.123)			
True			-0.239***	-0.236***		-0.238***	-0.235***
			(0.0360)	(0.0351)		(0.0359)	(0.0350)
True X Treatment			0.0102	0.0108		0.0106	0.0112
			(0.0451)	(0.0443)		(0.0451)	(0.0443)
Serial No.					-0.0108	-0.0238	-0.0236
					(0.0186)	(0.0158)	(0.0158)
Treatment X Serial No.					-0.0169	-0.0213	-0.0212
					(0.0279)	(0.0242)	(0.0242)
Constant	12.41***	13.35***	19.12***	22.34***	12.90***	17.99***	21.21***
	(2.565)	(2.483)	(2.851)	(7.913)	(2.470)	(2.765)	(7.897)
Observations	5,740	5,740	5,740	5,740	5,740	5,740	5,740
R-squared	0.073	0.073	0.213	0.222	0.073	0.212	0.221
Session FE	YES	YES	YES	YES	YES	YES	YES
Controls	NO	NO	NO	YES	NO	NO	YES
F	34.43	29.52	38.96	29.87	29.55	39.15	29.95

Notes: Dependent variable *Difference* in absolute value. Robust standard errors clustered on the individual level in the parentheses. *Round No* indicates the order number of a round in a session; *Serial No.* indicates the order number of a decision in a session.*** p<0.01, ** p<0.05, * p<0.1

Table A12: Regression analysis with controlling for risk-preferences.

	(1)	(2)	(3)	(4)	(5)	(6)	(7)	(8)
Signals:	Private		Public		Private		Public	
Dependent variable	Decision				Difference in absolute values			
Treatment	1.077	1.119	1.053	1.196	0.982	0.763	0.398	0.186
	(1.732)	(1.770)	(1.576)	(1.559)	(1.944)	(1.981)	(1.373)	(1.399)
Round after TSST	-0.482	-0.430	-1.706	-1.452	0.259	0.121	-1.905**	-1.921*
	(1.286)	(1.348)	(1.071)	(1.115)	(1.345)	(1.416)	(0.944)	(0.992)
Treatment X Round after TSST	-1.264	-1.312	0.779	0.458	-2.044	-1.892	0.328	0.351
	(1.489)	(1.547)	(1.307)	(1.345)	(1.573)	(1.634)	(1.184)	(1.221)
True	0.619***	0.619***	0.263***	0.261***	-0.115*	-0.126*	-0.339***	-0.344***
	(0.0551)	(0.0570)	(0.0245)	(0.0250)	(0.0624)	(0.0650)	(0.0363)	(0.0374)
True X Treatment	0.0243	0.0233	0.0217	0.0168	-0.0471	-0.0352	-0.0338	-0.0277
	(0.0724)	(0.0744)	(0.0323)	(0.0328)	(0.0752)	(0.0770)	(0.0484)	(0.0492)
True X Round after TSST	0.0293	0.0297	0.129***	0.127***	-0.00730	0.000320	0.00606	0.00388
	(0.0446)	(0.0461)	(0.0237)	(0.0242)	(0.0523)	(0.0547)	(0.0299)	(0.0312)
True X Round after TSST X Treatment	0.0147	0.0143	-0.0386	-0.0330	0.110*	0.102	0.0206	0.0224
	(0.0532)	(0.0545)	(0.0311)	(0.0313)	(0.0624)	(0.0644)	(0.0373)	(0.0383)
Certainty equivalent		10.81***		16.00***		-0.0765		-1.398
		(3.844)		(4.283)		(2.720)		(2.235)
Female							1.052*	
							(0.623)	
Constant	7.271	1.576	21.33*	13.64	13.81*	12.66	36.27***	36.76***
	(9.230)	(8.615)	(10.88)	(10.52)	(7.850)	(7.714)	(6.434)	(6.252)
Observations	5,320	5,168	3,920	3,808	5,320	5,168	3,920	3,808
R-squared	0.525	0.531	0.280	0.293	0.085	0.088	0.299	0.303
Session FE	YES	YES	YES	YES	YES	YES	YES	YES
Controls	YES	YES	YES	YES	YES	YES	YES	YES
F	32.47	31.02	38.49	35.49	2.397	2.355	19.06	20.67

Note: Dependent variables *Decision* (cols. 1-4) and *Difference* in absolute value (cols. 5-8). Robust standard errors clustered on the individual level in the parentheses. Certainty equivalent is calculated from an incentivized risk-elicitation task and may introduce endogeneity into estimation since it could be affected by treatment. It is rescaled to lie between 0 and 1 where 0.5 indicates risk-neutrality, lower values indicate risk-aversion and higher values indicate risk-seeking. *** p<0.01, ** p<0.05, * p<0.1

Table A13: Regression analysis with standardized variables for calculation of MDE.

	(1)	(2)	(3)	(4)
VARIABLES	Decision - standardized			
Treatment	0.0315	0.0315	0.0747	0.0856
	(0.0779)	(0.0739)	(0.0719)	(0.0628)
Round after TSST	0.0650	0.0650	0.0650	0.0330
	(0.0431)	(0.0432)	(0.0432)	(0.0384)
Treatment X Round after TSST	-0.00271	-0.00271	-0.00271	-0.0416
	(0.0564)	(0.0564)	(0.0565)	(0.0484)
Public	0.161***	0.161***	0.161***	0.123***
	(0.0384)	(0.0385)	(0.0385)	(0.0378)
Public X Treatment	0.0188	0.0188	0.0188	0.000942
	(0.0551)	(0.0551)	(0.0551)	(0.0541)
Public X Round after TSST	0.0441	0.0441	0.0441	-0.000548
	(0.0424)	(0.0424)	(0.0424)	(0.0417)
Public X Round after TSST X Treatment	-0.000882	-0.000882	-0.000882	0.0410
	(0.0556)	(0.0557)	(0.0557)	(0.0536)
True - standardized				0.468***
				(0.0366)
True std X Treatment				0.0302
				(0.0476)
True std X Round after TSST				0.180***
				(0.0326)
True std X Round after TSST X Treatment				-0.0323
				(0.0399)
Constant	-0.138**	-0.217	0.596	0.684
	(0.0549)	(0.152)	(0.509)	(0.528)
Observations	9,240	9,240	9,240	9,240
R-squared	0.011	0.024	0.043	0.379
Session FE	NO	YES	YES	YES
Controls	NO	NO	YES	YES
F	14.52	7.943	6.116	41.76
MDE (Treatment * Round after stress)	**0.158**	**0.158**	**0.159**	**0.136**

Note: Robust standard errors in parentheses. Mean Detectable Effect (MDE) calculated as 2.8 * SE of coefficient of interest. *** p<0.01, ** p<0.05, * p<0.1

Section B - discussion of the role of risk-preferences

This part is devoted to the discussion of the influence of risk-preferences on the relative weight of the signals in the decision-making procedure. The subjects were paid according to the quadratic scoring rule which is incentive compatible only for risk-neutral preferences. In the measurement of risk-preferences I obtained a wide variety of estimates of individual risk-aversion, which calls the employed payment scheme into question in terms of incentive compatibility. It is not clear whether this poses negative impact on the main results (treatment differences) since the procedure was constant across the two groups. A problem may arise when the risk-aversion interacts with treatment [83]. The subjects that become more risk-averse in treatment group may then face different incentives than the subjects in the control group: they should generally state their estimates of probability closer to the safe midpoint relative to the control group which would serve as a confounding factor. It is not clear though whether more risk-averse subjects should put more or less weight on the public signal relative to private, but generally this change in behavior due to change in risk-attitudes would be observed in the dif-in-dif regressions, though with no differences between reactions to public and private signals. I do not observe any significant differences in behavior between treatment and control groups in any of the steps of analysis, which may indicate either that the reaction to signals did not really depend on risk-preferences (maybe rather on ambiguity aversion) or there were two opposing forces: increased risk-aversion decreased the weight of both private and public signals while increased reward responsiveness under stress cancelled the effect on behavior mediated by the change in risk-attitudes [77]. Even though I do dispose with the individual risk-parameters (except for four subjects), I should not enter it as a control variable in the regression equation, because it is also determined by treatment and it thus raises concerns of potential bias stemming from the endogeneity of the relationship. Having this limitation in mind, I perform another robustness check to examine the stability of the coefficient of interest when I add the variable *Certainty equivalent* that represents the individual risk-attitudes into the three main regression

specifications. The results are presented in Table S12 where it is evident that indeed the coefficients of interest are fairly stable in terms of magnitude as well as their significance.

Section C – output from G_Power 3.1

t tests – Linear multiple regression: Fixed model, single regression coefficient

**Analysis:**	Sensitivity: Compute required effect size		
**Input:**	Tail(s)	=	One
	α err prob	=	0.05
	Power (1−β err prob)	=	0.8
	Total sample size	=	140
	Number of predictors	=	4
**Output:**	Noncentrality parameter δ	=	2.4990297
	Critical t	=	1.6562191
	Df	=	135

Effect size f² = 0.0446082

t tests – Linear multiple regression: Fixed model, single regression coefficient

**Analysis:**	Sensitivity: Compute required effect size		
**Input:**	Tail(s)	=	Two
	α err prob	=	0.05
	Power (1−β err prob)	=	0.8
	Total sample size	=	140
	Number of predictors	=	4
**Output:**	Noncentrality parameter δ	=	2.8217088
	Critical t	=	1.9776923
	Df	=	135

Effect size f² = 0.0568717

t tests – Linear multiple regression: Fixed model, single regression coefficient

**Analysis:**	Sensitivity: Compute required effect size		
**Input:**	Tail(s)	=	Two
	α err prob	=	0.05
	Power (1−β err prob)	=	0.8
	Total sample size	=	140
	Number of predictors	=	13
**Output:**	Noncentrality parameter δ	=	2.8231616
	Critical t	=	1.9789706
	Df	=	126

Effect size f² = 0.0569303

Section D – Details of Calculation of the Optimal Decisions in the Probabilistic Task – Variable *True*

For the sake of simplicity I assume that all subjects are rational and have symmetric expectations about other subjects which allows me to calculate the optimal response based on the information contained in the signals using the Bayes formula.

$$P(B|A) = \frac{P(B \cap A)}{P(A)}$$

For the value of $True$ after a certain number of signals, I assume all information a subject had received prior to the current decision in a given round was taken into account, while the preceding rounds were ignored. I also assume that a decision maker is rational and disregards any irrelevant information in the sense that there is no interdependence between the answers in the "What-if" scenario. Further, I assume that for calculating the public information, subjects also took into account the possibility that when the other subjects stated exactly the same probabilities of both bags having been selected, the resulting signal was chosen at random. For the particular calculation of each value of True we first looked at the composition of the balls in the bags. Let's define b the number of blue balls in the Blue bag, y the number of yellow balls in the Yellow bag, and n the total number of the balls in the bag. Note that $\Pr(b|B) = \Pr(y|Y)$, which denotes the probability of drawing the blue ball out of a Blue bag and drawing the yellow ball from the Yellow bag, respectively.

The optimal move after the first signal, which was always private, is calculated as follows: First the probability of drawing a blue ball given the composition is calculated: $\Pr(b|B) = b/n$, the probability of drawing the yellow ball is complementary, i.e. $\Pr(y|B) = 1 - b/n$. The value of $True$ is however the

probability of the bag being Blue, given this signal, which is $\Pr(B|b) = \frac{\Pr(b|B)}{\Pr(b|B)+\Pr(y|B)}$. The second signal could still be only a private one; it was the second draw with replacement from the same chosen bag. The optimal reaction is then calculated in the following way: first I need the chance each type of a signal composition could have appeared: $\Pr(bb|B) = \Pr(yy|Y) = \Pr(b|B)^2$; $\Pr(yb|B) = \Pr(yb|Y) = \Pr(by|B) = 2\Pr(b|B)\Pr(y|B)$ and $\Pr(bb|Y) = \Pr(yy|B) = \Pr(y|B)^2$. To calculate the values of *True*, I used these in the following fashion: $\Pr(B|bb) = \frac{\Pr(bb|B)}{\Pr(bb|B)+\Pr(by|B)}$, $\Pr(B|yy) = \frac{\Pr(yy|B)}{\Pr(yy|B)+\Pr(by|B)}$ and $\Pr(B|by) = \frac{\Pr(by|B)}{\Pr(by|B)+\Pr(yb|B)} = 50\ \%$. In the similar way, the rest of the private signals was calculated, when there were three or more private draws with replacement.

The first public signal could come at the earliest after two private ones. It was presented as a stylized bag indicating the color of the bag that the other player stated to be more probable. Let's label the blue bag presented as the decision of another participant as "B". If there was only one bag presented, it means that this other participant had received either (bb), or (by) and there was a 50 % chance of choosing B. Then $\Pr("B"|B) = \Pr("Y"|Y) = \Pr(bb|B) + 1/2\Pr(yb|B)$ and $\Pr("B"|Y) = \Pr("Y"|B) = \Pr(bb|Y) + 1/2\Pr(yb|Y)$. From this I derive that the probability of observing blue in both private signals as well as in the bag of another participant: $\Pr(bb"B"|B) = \Pr(bb|B)\Pr("B"|B)$ and similarly the probabilities of other events that could have happened: $\Pr(bb"B"|Y) = \Pr(bb|Y)\Pr("B"|Y)$; $\Pr(yy"B"|Y) = \Pr(bb"Y"|B) = \Pr(yy|Y)\Pr("B"|Y)$; $\Pr(yy"B"|B) = \Pr(bb"Y"|Y) = \Pr(yy|B)\Pr("B"|B)$; $\Pr(yb"B"|B) = \Pr(yb"Y"|Y) = \Pr(yb|B)\Pr("B"|B)$ and $\Pr(yb"B"|Y) = \Pr(yb"Y"|B) = \Pr(yb|Y)\Pr("B"|Y)$. These are necessary for the derivation of the probabilities that a chosen bag was blue, given the signals. If the private signals were both blue (*bb*) and the other participant's bag was also blue ("B"), then $\Pr(B|bb"B") = \frac{\Pr(bb"B"|B)}{\Pr(bb"B"|B)+\Pr(bb"B"|Y)}$ and similarly if the observed other participant's bag was yellow: $\Pr(B|bb"Y") = \frac{\Pr(bb"Y"|B)}{\Pr(bb"Y"|B)+\Pr(bb"Y"|Y)}$. If both private signals were yellow, we get the

following for other participant's bag being blue and yellow, respectively: $\Pr(B|yy\text{"Y"}) = \frac{\Pr(yy\text{"}Y\text{"}|B)}{\Pr(yy\text{"}Y\text{"}|B) + \Pr(yy\text{"}Y\text{"}|Y)}$ and $\Pr(B|yy\text{"B"}) = \frac{\Pr(yy\text{"}B\text{"}|B)}{\Pr(yy\text{"}B\text{"}|B) + \Pr(yy\text{"}B\text{"}|Y)}$. For the case of a mixed private signal, (yb), the public signal is : $\Pr(B|yb\text{"B"}) = \frac{\Pr(yb\text{"}B\text{"}|B)}{\Pr(yb\text{"}B\text{"}|B) + \Pr(yb\text{"}B\text{"}|Y)} = \Pr(\text{"}B\text{"}|B)$. In the same fashion, I calculate the optimal probabilities for more private and public signals.
