# Supplementary material for: Social learning under acute stress
Source: PLoS One. 2018 Aug 22;13(8):e0202335. doi: 10.1371/journal.pone.0202335 (PMC6104985; doi:10.1371/journal.pone.0202335)
Supplement: S2 File — This file contains a detailed description of the treatment procedure, including a scheme of the room, the script and instructions for both treatment and control groups, and the text used for the debriefing of the treatment group. (PDF) [file pone.0202335.s002.pdf]

TSST-G Protocol

Disclaimer: this is a slightly modified version of a protocol that was developed by Bernadette Von Dawans. When used, the resulting publication must include the citation of the original article this script was developed for:

von Dawans, B., Kirschbaum, C., & Heinrichs, M. (2011). The Trier Social Stress Test for Groups (TSST-G): A new research tool for controlled simultaneous social stress exposure in a group format. *Psychoneuroendocrinology*, *36*(4), 514–22. doi:10.1016/j.psyneuen.2010.08.004

Content:

1. TSST-G room setting..2
2. Experimental script – treatment group....................................3
3. Experimental script – control group......................................7
4. Experimental instructions – treatment group..............................9
5. Experimental instructions – control group................................10
6. Debriefing – treatment group...11

1. **TSST-G room setting (treatment condition)**

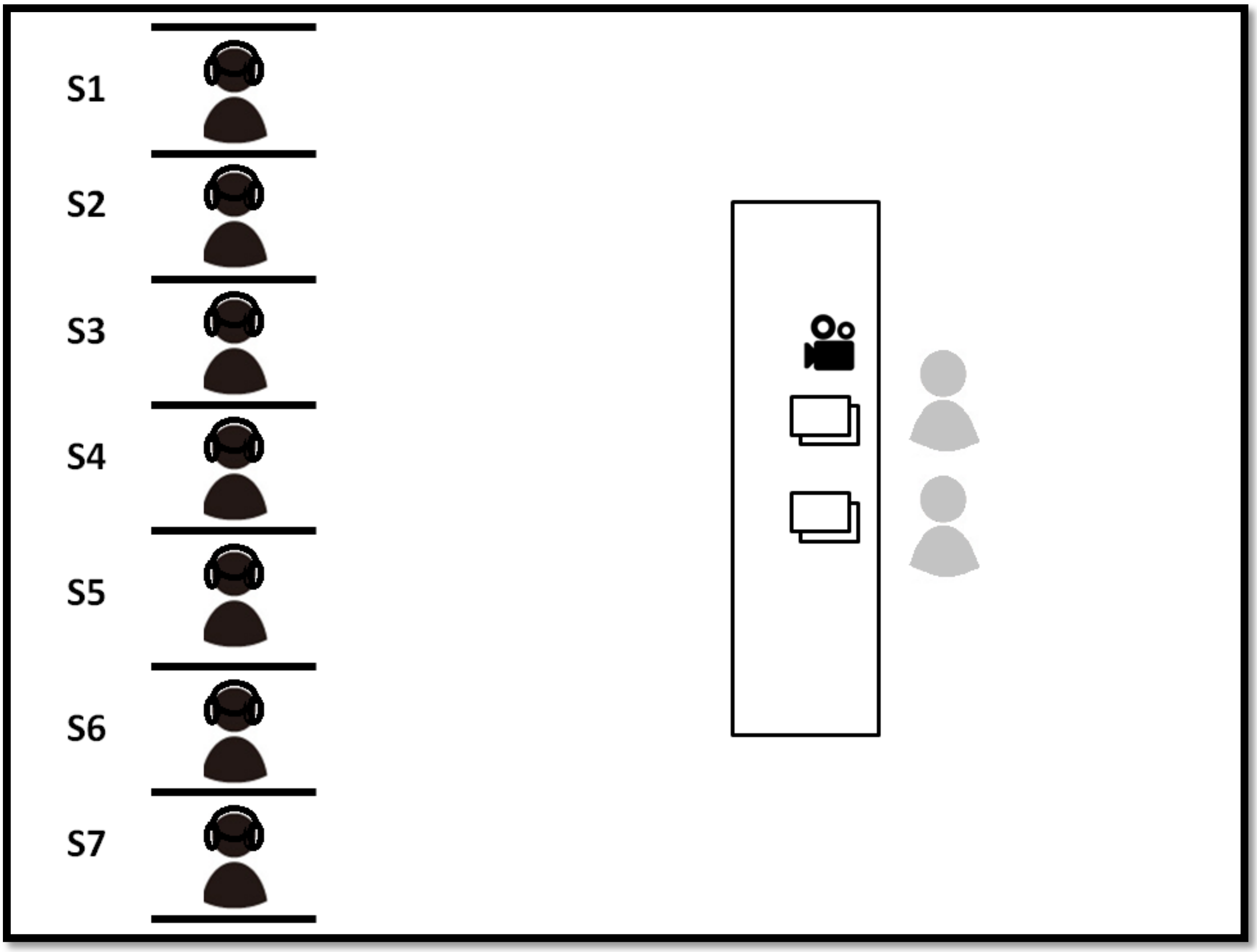

2. Experimental script – treatment group

Heart rate marker

1. Job Interview

The participants are led into the TSST-room by the Experimenter and given a verbal introduction to the task by the Experimenter.

Each of you will now have 2 minutes to present yourself to the panel. To avoid hearing each other, you will be asked to take headphones on when not speaking to the panel. The panel will call you by showing your participant number. When you see your number being shown, please take off your headphones and start with your interview. After you finish, please take the headphones back on. You will be recorded by a camera. The members of the panel will observe your behavior closely and will take notes during your talk. <u>They may ask you follow-up and clarification questions, even during the interview of another person.</u> Following your talk you will be given a second task, which will be explained to you then by the panel. <u>You will be called on in random order and may be called on again at any time.</u> Do you have any questions?

After the camera is turned on and all participants are standing at their correct locations, the Experimenter requests that they all press on the heart rate marker button.

- **Please press the red button on your watch now.**

Heart rate marker

The Experimenter then leaves the room, and the chairperson of the panel begins to call on participants randomly by their participant numbers and asks them to begin their speech.

- **Please take on the headphones.**

- **Number X: Please begin!** Time: 00:00

Only the chairperson should address the research participant directly, so that coordination problems between the panel members can be avoided. The panel should let the research participant speak freely. In most cases the participant will come to the end of the talk before two minutes have passed. The panel should then give him/her time to continue. In this case, there should be a pause. The pause is important to elevate the participant's stress.

After a ten to twenty-second pause, the chairperson can alert the research participant to the remaining time, as with the phrase **"You still have time, please continue...".** *Should it appear after another ten seconds that the participant has nothing further to say, then the chair should ask questions until the end of the time period. The phrasing of these questions is left to the chair's discretion; they may also be based on the participant's previous statements*.

Typical questions in this context:

- **Why do you think you are especially well-qualified for this task?**
- **Why do you think you are better qualified then the other applicants?**

- **How would your family and friends describe you?**
- **What qualities do you value in a friend?**
- **What qualities do you value in a co-worker?**
- **You just mentioned that you have a particular talent for ____. What other special talents would you say that you possess?**
- **You were just speaking about ____. What do you think about ____?**
- **Please complete this sentence: "I am the best at...."**
- **Describe your leadership abilities.**
- **What are your long-term career goals?**
- **What is your opinion about teamwork?**

In some instances the research participant will speak calmly and fluently for first full minute. In this case, the chair wants to intervene between the first and second minute to ask questions to the research participant. This should also be dependent on what is being said by the participant and his/her stress level. For instance, it is not appropriate for the applicant to speak in great detail about specific lessons learned in the course of training at university or elsewhere. Some research participants use their school-knowledge to avoid describing themselves. In that case the chair should certainly intervene, for example by saying "We believe that you know how to execute a market analysis, but we would be more interested to find out why you were so involved in or drawn to this area."

Other typical interventions in this context:

- **Thank you, but we would prefer to hear more about your strengths.**
- **Thank you, but we would prefer to hear more about your problem solving abilities.**

(The participants were asked to stay straight and with their hands next to their body when delivering the interview. In the case when the participants try to ease themselves by changing their posture, you may remind them not to do so.

- **Please, stay straight.**
- **Please, take your hands out of your pocket.**)

After two minutes, the first participant is interrupted and the next participant is randomly selected and called by showing his/her number. The same procedure is followed until all participants have completed the task. The total time should be 14 minutes.

- **Thank you—that's enough.** Time: 14:00

Ask all participants to take the headphones off.
Please press the red button on your watch.

Heart rate marker

2. Mental arithmetic task

It is the <u>chair</u>'s duty to explain the second part of the stress protocol. All participants should take off their headphones to hear the instructions, then they should take them back on.
A typical transition would sound like this:

We now want you to solve a calculation task. Please count aloud backwards from the number we give you to zero in 17-step sequences. For example, if you are given number 102, you should calculate 102 – 85 – 68 – 51 and so on. Please calculate as quickly and correctly as possible. Should you miscalculate, we will point out your mistake and either you or another participant will have to try again or start over. We will call on you with your participant number. You may also be called on multiple times. Please take the headphones back on.

Call the participants by showing their number: **Please begin now with the number ___. (Please count aloud backwards from the number we give you to zero in 17-step sequences)**

Time: 16:00

Each participant is given his or her own starting number, as can be seen below (for example, participant 5's starting number is 4674) and should compute for about a minute. This list should be available to the panel members so that they do not have to calculate the numbers themselves.

If the research participant miscalculates, the chair should respond with the standard phrase:

- **That is incorrect. Please try again.** *(two times)* **Please begin again with ____.**

If the participant is calculating slowly/not making any mistakes: **Please calculate faster (speak louder).**

End with: **Thank you – that's enough. Please, put on the headphones.**

1: 4878	→	4861	→	4844	→	7: 4827	→	4810	→	4793	→	6: 4776
4759	→	2: 4742	→	4725	→	4708	→	4691	→	5: 4674	→	4657
4640	→	4623	→	3: 4606	→	4589	→	4: 4572	→	4555	→	4538
4521	→	4504	→	4487	→	4470	→	4453	→	4436	→	4419
4402	→	4385	→	4368	→	4351	→	4334	→	4317	→	4300
4283	→	4266	→	4249	→	4232	→	4215	→	4198	→	4181
4164	→	4147	→	4130	→	4113	→	4096	→	4079	→	4062
4045	→	4028	→	4011	→	3994	→	3977	→	3960	→	3943
3926	→	3909	→	3892	→	3875	→	3858	→	3841	→	3824
3807	→	3790	→	3773	→	3756	→	3739	→	3722	→	3705
3688	→	3671	→	3654	→	3637	→	3620	→	3603	→	3586
3569	→	3552	→	3535	→	3518	→	3501	→	3484	→	3467
3450	→	3433	→	3416	→	3399	→	3382	→	3365	→	3348
3331	→	3314	→	3297	→	3280	→	3263	→	3246	→	3229

3212	→	3195	→	3178	→	3161	→	3144	→	3127	→	3110
3093	→	3076	→	3059	→	3042	→	3025	→	3008	→	2991
2974	→	2957	→	2940	→	2923	→	2906	→	2889	→	2872

- **Thank you—that's enough.** Time: 30:00

Ask the participants to take the headphones off.
Please press the red button on your watch.

heart rate marker

- **This is the end of this task. The experimenter will take you back to the lab now. Please remember that you still may not speak to each other.**

The chairperson opens the door, and the experimenter (who should be waiting outside) leads the participants back to the laboratory.

3. Experimental script – control group

Heart rate marker

→ NO CAMERA

→ NO LAB COATS

1. Reading task

The participants are led into the TSST-room by the Experimenter and given a verbal introduction to the task by the Experimenter.

Your task in this experiment to read the given text aloud. Your reading and English ability will NOT be assessed during this task. Your group will read out loud for a total of 14 minutes, but you may take small breaks during this period and are free to choose your own reading speed. Speak just loudly enough so that your voice can be heard. You are not required to speak so loudly that all the other participants can hear you. The two-person panel will not be judging you; they will simply provide instructions. Following this task you will be given a second task, which will be explained to you then by the panel. The second task is also not a test of your abilities, but consists rather of a simple cognitive task. Do you have any questions?

After all participants are standing at their correct locations, the Experimenter requests that they all press on the heart rate marker button.

- **Please press the red button on your watch now.**

heart rate marker

The Experimenter then leaves the room, and the chairperson of the panel requests that the participants begin.

- **Please begin.** Time: 00:00

The participants should now all read the text aloud—not too loudly. A general "babble" should be heard in the room. During this time, the panel should occupy themselves with their own papers and should NOT observe the participants. If the panel notices that a participant is not reading at all, the chairperson should ask him or her to continue reading.

- **Thank you—that's enough.** Time: 14:00

Please press the red button on your watch.

Heart rate marker

2. Counting task

We now would like you to do a counting task using specific sequences of numbers. We will begin with counting aloud by multiples of two (in other words, 2, 4, 6, and so on). We will

inform you when it is time to switch to a different sequence of numbers. This task is also NOT a test of ability and you will NOT be evaluated. Please begin now.

Time: 16:00

The panel asks the participants to count by multiples of 2, 5, 10, 20, and so on – the sequences should be changed every couple minutes or so, to prevent the participants from becoming bored.

Please switch to counting by multiples of five (in other words, 5, 10, 15, 20, and so on).

- **Thank you, that's enough.** Time: 24:00

Please press the red button on your watch.

Salivette, heart rate marker

- **This is the end of this task. The experimenter will take you back to the lab now. Please remember that you still may not speak to each other.**

The chairperson opens the door, and the Experimenter (who should be waiting outside) leads the participants back to the waiting room.

4. Experimental instructions – treatment group

Your task is the following: Please imagine that you applied for a job and have been invited for an interview. In contrast to a real interview, however, you are required to give a talk, in which you are to convince the panel in two minutes that you are the best candidate for this position.

You should primarily focus on your personal qualities—in other words, the personality traits that distinguish you from other applicants and qualify you for this position. You should not focus on your knowledge or professional qualifications—assume that the panel has already received information about your academic and professional background. The members of the panel will observe your behavior closely and will take notes during your talk. Please note that you will also be recorded by a camera. You should try to leave the best possible impression, and assume the role of the applicant for the duration of the talk as best as you can. The panel may ask you follow-up and clarification questions, even during the interview of another person.

Note that you should stay straight and with your hands next to your body for the whole time of the task.

Following your talk you will be given a second task, which will be explained to you then by the panel. You will be called on in random order and may be called on again at any time. The entire procedure will last approximately 30 minutes. Please note your participant number (the number of your computer terminal). The panel will call on you using this number.

During the time when other participants speak, you will be asked to put on headphones to avoid hearing the other participants. The headphones will be on the table behind you. After the first task finishes, the chairperson of the panel will ask you to put the headphones off to hear further instructions all together.

When you have finished reading these instructions, please take the sticker with your participant number and place it on your chest. You now have 3 minutes to prepare. You may take some notes now, but you may not use them during your talk.

If you have any questions, please raise your hand and we will come to your seat. Please, do not communicate with other participants.

5. Experimental instructions – control group

Your task will be to read a text aloud. In a few minutes, you and the other participants will be asked to simultaneously read the given text aloud. Your reading and English ability will not be assessed during this task.

Your group will read aloud for a total of 14 minutes, but you may take small breaks during this period and are free to choose your own reading speed. Speak just loudly enough so that your voice can be heard. You are not required to speak so loudly that all the other participants can hear you. The two-person panel will not be judging you; they will simply provide instructions.

Following this task you will be given a second task, which will be explained to you then by the panel. The second task is also not a test of your abilities, but consists rather of a simple cognitive task. The entire procedure will last approximately 30 minutes. Please note your participant number (the number of your computer terminal). The panel will call on you using this number.

When you have finished reading these instructions, please take the badge with your participant number and place it on your chest. You now have 3 minutes to prepare.

If you have any questions, please raise your hand and we will come to your seat. Please, do not communicate with other participants.

6. Debriefing – treatment group

Thank you for participating in the experiment. Before we proceed to the payments, we would like to say few things regarding Part 2 of the experiment – the challenge task you went through in the adjecent room.

Part 2 consisted of a standardized procedure, with the purpose to induce mild stress on you. The behavior of the panel in no way reflected what you were saying - the panel members were instructed to give absolutely no feedback and to act completely neutrally. Your behavior in the second part will not be analyzed in any way and the video recording will shortly be destroyed. (You may perceive the procedure as a good training for example for language exams, where the panel is also instructed to not give any feedback).

If you have any other questions regarding the challenge task, please stay here after the payments and we will talk to you then.

We need to ask you not to talk about any part of the experiment with anyone. This is absolutelly essential for not spoiling our future results. This is why you are asked to sign a confidentiality statement, which we will now go through together:

Confidentiality statement

We would like to inform you that an essential prerequisite for the implementation of the experiment you have experienced today is the novelty of the situation. The scientific success of further studies depends essentially on the assumption that the situation is new and unknown to the study participants. We would therefore like to ask you not to describe any part of the experiment to potential future participants – that would be friends, classmates and so on.

I herewith declare that, in the interest of the development of scientific understanding, I will not pass on any information about the experiment to friends and acquaintances.

Date: Signature:
