# Supplementary material for: Social learning under acute stress
Source: PLoS One. 2018 Aug 22;13(8):e0202335. doi: 10.1371/journal.pone.0202335 (PMC6104985; doi:10.1371/journal.pone.0202335)
Supplement: S3 File — This file contains the instructions that were given to subjects about the task measuring the updating behavior that is described in the main text in section 3.1. (PDF) [file pone.0202335.s003.pdf]

EXPERIMENTAL INSTRUCTIONS

Task

There are two unmarked bags, which are filled with colored balls, blue and yellow. The bags are different in the number of balls of each color. The “Yellow” bag always has more yellow balls than blue balls and the “Blue” bag always has more blue than yellow balls. For example, “Yellow” bag can contain 5 yellow and 4 blue balls; then the “Blue” bag will contain 4 yellow and 5 blue balls. The computer will secretly pick one of the two bags with 50/50 chances to select each bag. Your goal in this experiment is to correctly say in each round which of the two bags was chosen, or merely state how sure you are about it. To help you find out which bag was chosen, you will get a number of signals, which we will describe later.

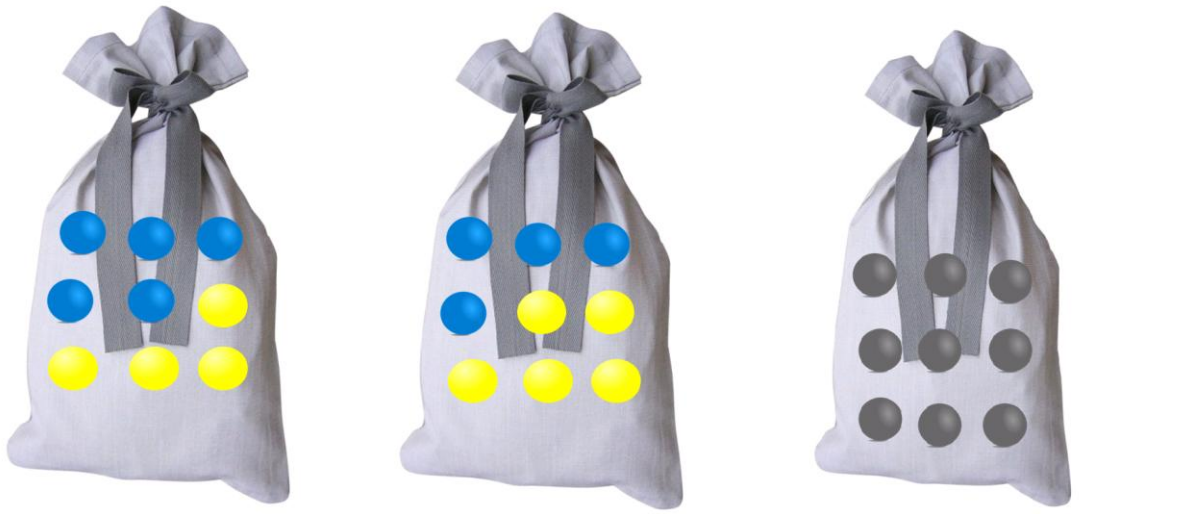

Figure 1

The numbers of yellow and blue balls in the bags will be different across rounds - you will see before each round the exact number of blue and yellow balls in the two bags. Computer selects one of the bags at the beginning of the round. Each round then consists of several steps, where you will be given a new signal about which bag was chosen. In each step, you will be asked to report your beliefs regarding which bag was chosen. After you make all decisions, the round is over and you will see which bag was really chosen. The experiment then proceeds to a next round.

Private signal

First, you will be given a private signal. The computer draws a ball for you from the chosen bag and shows you its color. Then the ball is put back into the bag and the computer shakes with it before the ball is drawn for a next person. Each participant receives his/her own signal privately – for each participant the computer will draw separately, but from the same chosen bag for all. You will see the color of your private signals in the top-left corner of the screen (see Figure 2). You will never learn private signals of anyone else. The number of private signals is also different across rounds.

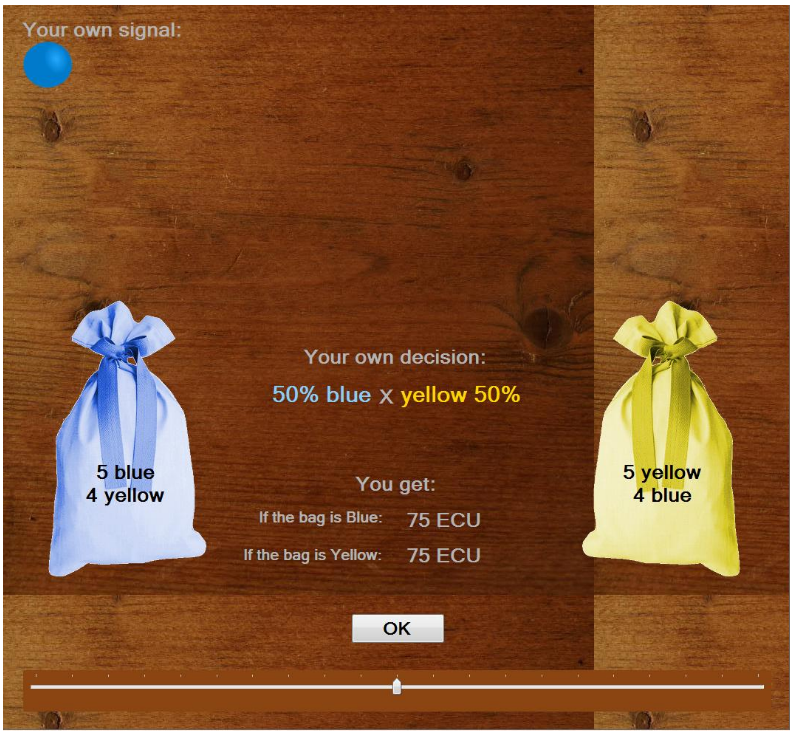

Figure 2

After you see your private signal, you will be asked to report what you think the chances are that you are drawing from either bag. For this, please use the slider that you will see in the bottom part of the screen (see Figure 2). The position of the slider determines the probability that you think the bag is "Blue" or "Yellow". The exact probabilities set by the slider can be seen in the middle of the screen and they change as you move with the slider. Right above the slider you will see the amount of ECU that you get if the chosen bag is indeed "Blue" or "Yellow". Again, these numbers change as you change your stated probabilities (as you move the slider). Feel free to move the slider back and forth to see the effects on earnings until you are happy with your choice. You can also refer to Table 1 below which presents the payment function (the link between stated probabilities and earnings) for selected probabilities. Click OK to confirm your choice.

Information about the decisions of others 1 – "Reality"

After all private signals have been revealed, you will also see information about the decisions of some other participants. It will appear in the top right corner and it will look like bags of blue or yellow color (see Figure 3). If the bag shown is blue, it means that the participant set his/her slider such that the probability of the bag being Blue is higher than 50%. If the bag shown is yellow, it means that the participant set his slider such the probability of the bag being Yellow is higher than 50%. This information will be taken from the latest decision made by the participant, i.e. from the decision made in the very previous step. If a participant set the probabilities to 50/50, one color will be randomly chosen. After you see this, you can again reposition the slider to state your beliefs about which bag is being drawn from. This situation will be labeled with a sign "Decisions of others: Reality" to the right of the colored bags stating the decisions of others.

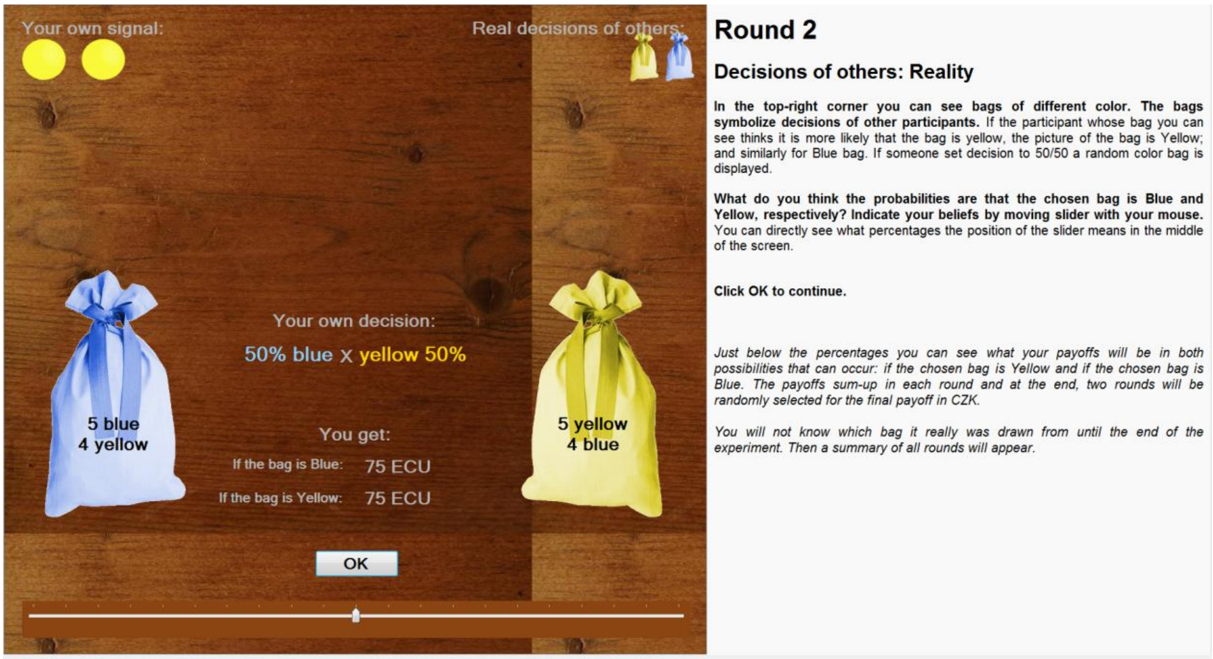

Figure 3

Information about decisions of others 2 – "What if…"

In some cases we will not show you directly what the other participants set. Rather we will show you all possible combinations of what they could have set, one after another – different scenarios that could have happened. After each presented scenario you can reposition the slider again to set your beliefs about the chances that you are drawing from either bag. Then you will see which scenario really happened and you will be paid according to what you reported in this scenario. This situation will be labeled with a sign "Decisions of others: What if…" to the right of the colored bags stating the decisions of others.

Final Payment

Your payment from this part of the experiment depends on how correctly you can guess which bag was chosen by the computer. We have created the payment function in such a way that you should expect to earn the most money when you report your beliefs exactly as they are. By understating or overstating your beliefs you are not maximizing the amount of money you could make.

You will play several rounds of the game described above. The payoff in each round will be the sum of payoffs in all its steps – in other words, every time you set your beliefs about which bag was chosen matters within the given round. In the case of the "What if…" information type, only one scenario counts – the scenario that represents the real situation of the decisions of others. At the end of the experiment **three** rounds out of all will be randomly chosen for the payoff and the total payoff will be their sum. The selection will be made by drawing a number from a real bag in front of all participants. The payoff from the lottery task and your show-up fee will be added to the payoff from this task to determine your final payment.

Percent Chance of the Blue bag	Percent Chance of the Yellow bag	Your Earnings if we are drawing from the Blue Bag (in ECU)	Your Earnings if we are drawing from the Yellow Bag (in ECU)
100%	0%	1000	0
95%	5%	998	98
90%	10%	990	190
85%	15%	978	278
80%	20%	960	360
75%	25%	938	438
70%	30%	910	510
65%	35%	878	578
60%	40%	840	640
55%	45%	798	698
50%	50%	750	750
45%	55%	698	798
40%	60%	640	840
35%	65%	578	878
30%	70%	510	910
25%	75%	438	938
20%	80%	360	960
15%	85%	278	978
10%	90%	190	990
5%	95%	98	998
0%	100%	0	1000

Table 1

Trial round

Before the rounds are played for real, there will be two trial rounds for you to try-out the task. Trial rounds will not be paid for. Feel free to move the slider as much as you want to see the effect on the payment.

Round outline - summary

At the beginning of each round you will see a screen with the information about how many blue and how many yellow balls there are in each bag. The computer then randomly selects one of the bags. After that, you will receive private signals – computer draws one ball from the chosen bag only for you, shows you its color and puts it back again. Your task is to state your belief about which of the bags was chosen. When you position the slider where you want it, click ok and the next private signal may appear. After receiving all private signals in a round and positioning the slider in each case, information about the behavior of others will appear. You may change the position of the slider again. After clicking OK, the round is over. When a round is over, you will find out what the color of the chosen bag was. You will see the summary of results of all rounds at the end of the experiment.

Extra task

After some rounds, this task will be interrupted by the extra task, which will be performed in another room. When you get back, you will continue with some more rounds of this task.

Help

The most important part of the instructions will always be on the right half of the screen as a help for you. Most of it stays the same during the whole experiment.

Now, you will be asked to answer some control questions to make sure you understand the instructions (not to cancel your participation). If you have any questions, please raise your hand and the experimenter will come to you.
