# Supplementary material for: Social learning under acute stress
Source: PLoS One. 2018 Aug 22;13(8):e0202335. doi: 10.1371/journal.pone.0202335 (PMC6104985; doi:10.1371/journal.pone.0202335)
Supplement: S4 File — This file includes the exact wording of the control questions that were checking the understanding of subjects to the rules of the main task. (PDF) [file pone.0202335.s004.pdf]

Control questions

Let say there are 6 yellow and 4 blue balls in the Yellow bag and 4yellow and 6 blue balls in the Blue bag.

1. Your task is to…

1) state in which bag is more yellow balls

2) state in which bag is more balls in total

3) state which bag was chosen by computer

4) state which color is more likely to be preferred by the experimenter

2. The bags in the top-right corner indicate…

1) How much you earned in the experiment

2) Number of lives you have left in the game

3) The decisions of other participants

4) Investment tokens

c. If you see in the top-right corner a blue bag, it is very likely that …

1) one other participant thinks it is more likely that the chosen bag is Blue

2) one other participant set the probabilities 50/50 and color yellow was randomly chosen

3) one other participant has not done any decision yet

4) one other participant has left the room

d. You will see how much ECU you earned in a particular round …

1) After each round

2) After each two rounds

3) After each three rounds

4) Never

e. If there is a sign "Decisions of others: Reality" in the top-right corner of the screen, it means that…

1) in the next round you will not be paid for your decisions

2) the colors of the bags next to the sign indicate what color the other participants think is more probable

3) the participants' heart rate is too high

4) nothing from above

f. The balls are being drawn …

1) for each participant from another bag

2) for each participant from the same bag in all rounds

3) for each participant from the same bag in the given round

4) from two different bags in a given round

g. If there is a sign "Decisions of others: What if" in the top-right corner of the screen, it means that you will see all scenarios …

1) of what really happened in different groups of participants in the lab

2) of what a selected group of other participants could have set and you will be asked to make your decision for each case.

3) of what the others could have set and will be asked to make your decision for only one case.

4) of what you will do in the next round.

g. If there is a sign "Decisions of others: What if" in the top-right corner of the screen, it means that you will be paid according to which scenario?

1) All scenarios will then count towards your payments.

2) Only the true scenario (the one that really happened) will count.

3) Only the wrong scenario will count.

4) Randomly chosen scenario will count.

g. Your payment from this part of the experiment will depend on

1) your decisions in all rounds, and every time you set your decision in a given round matters

2) your decisions in two randomly selected rounds, and every time you set your decision in a given round matters

3) your decisions in all rounds, and just your final decision in a given round matters

4) your decisions in two randomly selected rounds, and just your final decision in a given round matters
